# Interfacial Evolution and Accelerated Aging Mechanism for LiFePO_4_/Graphite Pouch Batteries Under Multi-Step Indirect Activation

**DOI:** 10.1007/s40820-025-01971-2

**Published:** 2026-01-05

**Authors:** Yun Liu, Jinyang Dong, Jialong Zhou, Yibiao Guan, Yimin Wei, Jiayu Zhao, Jinding Liang, Xixiu Shi, Kang Yan, Yun Lu, Ning Li, Yuefeng Su, Feng Wu, Lai Chen

**Affiliations:** 1 School of Materials Science and Engineering, Beijing Key Laboratory of Environmental Science and Engineering, Beijing Institute of Technology, Beijing, 100081 People’s Republic of China; 2 Chongqing Innovation Center, Beijing Institute of Technology, Chongqing, 401120 People’s Republic of China; 3 China Electric Power Research Institute, Haidian District, Beijing, 100192 People’s Republic of China; 4 21C Innovation Laboratory, Contemporary Amperex Technology Co., Ningde, 352100 People’s Republic of China

**Keywords:** Accelerated aging, Electrode/electrolyte interface, Multi-step segmented indirect activation, EEI film, Dissolve of Fe ions

## Abstract

The dissolution of iron from the cathode and electrode/electrolyte interface (EEI) during long cycles significantly accelerates the aging process of LiFePO_4_ (LFP)/graphite batteries; there is a lack of systematic understanding of the spatial distribution of the EEI interface layer and the dissolve of Fe ions, especially in terms of the mechanism of the cathode–electrolyte interphase (CEI), solid electrolyte interphase (SEI), and iron dissolution. In this study, aged cells were subjected to continuous activation with constant current and multi-step segmented indirect activation (IA) and analyzed for capacity fade, impedance growth, and active Li^+^ mass loss at the EEI and nanoscale levels. The interaction between dissolved Fe^2+^ and the EEI in LFP/graphite pouch batteries was proposed and verified. The findings indicate that during IA process, the electric field facilitates the migration of solvated ions toward the electrodes, while simultaneously inhibiting the formation of organic species such as ROCO_2_Li. The SEI primarily consists of a mixture of organic and inorganic small molecules, forming a continuous and uniform film on the electrode surface. This study demonstrates that IA favors the formation of a uniform EEI and offers constructive insights for advancing accelerated lifetime prediction strategies in lithium-ion batteries.

**Supplementary Information:**

The online version contains supplementary material available at 10.1007/s40820-025-01971-2.

## Highlights

- Quantifying the aging mechanisms and their evolution patterns during battery aging is crucial for enabling renewable energy.
- The uniform electrode/electrolyte interface (EEI) film on the electrode surface has an important impact on the energy density, cycling performance and power density of the battery.
- Multi-step segmented indirect activation strategy promotes the formation of uniform EEI and suppresses iron dissolved in the electrolyte.

**Supplementary Information:**

The online version contains supplementary material available at 10.1007/s40820-025-01971-2.

## Graphical abstract

**Figure:**
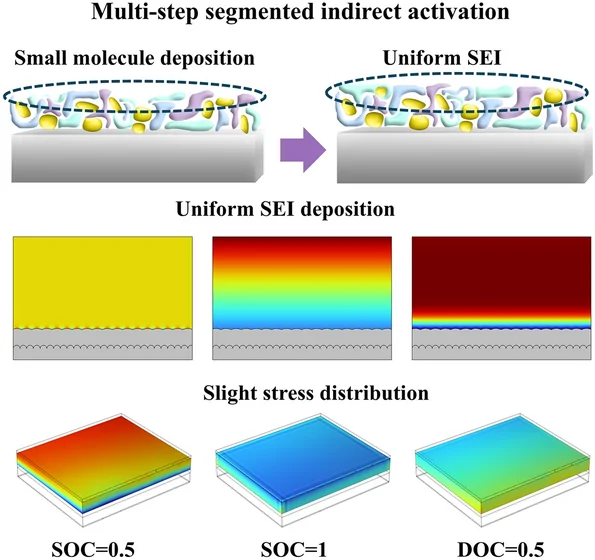

## Introduction

Lithium iron phosphate (LiFePO_4_, LFP) batteries have garnered significant attention in the field of lithium-ion batteries (LIBs) because of their high sustainability, superior thermal stability, extended cycle life, and cost-effectiveness [1–3]. Although LFP demonstrates exceptional structural stability and a high theoretical capacity of 170 mAh g^−1^, LFP/graphite batteries still suffer from capacity fading, impedance growth, metal dissolution, and material degradation over extended cycling. Additionally, direct contact between the electrode and electrolyte can induce unavoidable parasitic reactions, accompanied by ongoing surface structural reconstruction and the formation of passivation layers [4]. Concurrently, Fe ions irreversibly migrate into the Li layer in a highly attenuated state, resulting in cation mixing and phase transformation. These phenomena collectively compromise battery performance, increase polarization, and ultimately impede the design and scalability of LIBs. Therefore, performance degradation remains one of the foremost challenges to long-term operation, necessitating a comprehensive understanding of the mechanisms driving capacity loss and the development of effective mitigation strategies [5].

The electrolyte–electrode interphase (EEI) film and iron (Fe) dissolution are important incentives for accelerated aging in LFP batteries. Their interaction significantly impacts the battery cycle life, capacity fading, and safety performance, and the failure of the EEI is directly related to the aging of batteries [6]. Under ideal conditions, the EEI film gradually forms during the initial cycle and subsequently exists as a stable passivation layer throughout the entire service life. However, the formation of an EEI film involves a complex multistage electrochemical/chemical redox process, and the composition, structure, and function of EEI films also undergo dynamic changes during cycling. Concurrently, the formation of the EEI film consumes both the electrolyte and the active ions, potentially leading to losses in the accessible energy and/or power density of the battery [7].

The EEI, comprising both the cathode–electrolyte interphase (CEI) and the solid electrolyte interphase (SEI) at the anode, plays a pivotal role in determining the stability of the system, directly affecting electrolyte decomposition and the loss of active Fe [8, 9]. During prolonged lithiation/delithiation cycles, the organic outer layer of the CEI progressively thickens due to the oxidative decomposition of electrolyte components, such as Li_2_CO_3_ and ROCO_2_Li [10]. This continuous CEI growth increases lithium-ion diffusion resistance, thereby impeding the intercalation and deintercalation kinetics of Li^+^ within the cathode material. These processes collectively contribute to enhanced polarization and capacity degradation. The dissolution of Fe primarily arises from the structural breakdown of the cathode, while the migration of Fe^3+^ ions initiates secondary reactions that further catalyze electrolyte decomposition and promote the generation of unstable organic species within the SEI [11, 12]. On the anode side, the concurrent effects of SEI thickening and Fe deposition significantly elevate interfacial impedance and intensify polarization.

To date, systematic electrochemical analyses coupled with advanced characterization techniques have elucidated the formation processes of the EEI. However, the underlying correlations among the basic structure/composition, formation mechanism, Fe dissolution, and battery performance of EEI films remain poorly understood. Furthermore, the synergistic mechanisms by which the SEI and CEI collectively contribute to battery aging remain inadequately understood [13]. In this study, we conducted a quantitative analysis of accelerated aging mechanisms under varying activation conditions using a suite of multiscale characterization techniques, including time-of-flight secondary ion mass spectrometry (TOF-SIMS), focused ion beam (FIB) analysis, laser ablation inductively coupled plasma–mass spectrometry (LA-ICP-MS), and advanced XANES spectroscopy. Coupled with DFT calculations and COMSOL simulations of SEI evolution in pouch-type full cells for both cathodes and anodes, this approach enabled a comprehensive elucidation of the dynamic interfacial evolution at the EEI/Fe interfaces.

This work reveals that EEI failure and Fe dissolution synergistically accelerate battery aging through multiple pathways, including interfacial side reactions, catalytic decomposition, and impedance growth. The non-uniform growth of the SEI and CEI leads to deteriorated kinetic performance and irreversible phase transformations. These mechanisms collectively accelerate battery aging during cycling. This study demonstrates that multi-step segmented indirect activation (IA) at varying current densities facilitates the formation of thin, uniform SEI and CEI layers—composed of mixed organic and inorganic small molecules—on both cathode and anode surfaces. These interfacial films promote efficient lithium-ion transport and suppress phase transitions, thereby improving the cycling stability of the batteries. This finding offers valuable insights into the intricate relationship between electrode–electrolyte interfacial structures and battery performance, providing a foundation for future investigations in this domain.

## Experimental Section

### Materials Characterizations

The crystal structure data of all the samples were examined via Rint-2000 X-ray power diffraction within 10°–80° at a scan rate of 10° min^−1^ (the Rietveld refinement program at 2° min^−1^ by the General Structure Analysis System, GASA). By in situ X-ray diffraction test, a custom-made Swagelok battery with an X-ray transparent aluminum window was used to describe the cell mechanism. To analyze the morphology and microstructure of the samples, scanning electron microscopy (SEM; JEOL, JSM 6400) with a focused ion beam (FIB, FEI, SCIOS) was used; transmission electron microscopy (TEM; JEOL, 2100F), X-ray photoelectron spectroscopy (XPS, Thermo Fisher ESCALAB 250Xi), TOF-SIMS (ION-TOF GmbH TOF-SIMS), and X-ray absorption spectroscopy (XAS) were performed on beamline BL01C1 in the NSRRC to characterize the elemental composition, elemental valence state, and corresponding material structure at the surface and depth positions. Laser ablation inductively coupled plasma–mass spectrometry (LA-ICP-MS) was used to characterize the distributions of the corresponding elements. Swelling was monitored using an in situ swelling analyzer (IEST-SWE2100, Initial Energy Science & Technology Co., Ltd., IEST) with submicron resolution, under controlled conditions of 25 ± 1 °C in a thermostatic chamber to avoid thermal interference. To assess the overall mechanical environment of the material, a single-particle force tester (IEST, SPFT-2000) equipped with high-precision force and displacement sensors was used to measure the compressive strength of individual particles within a certain period of time.

### Electrochemical Tests

For one cell, continuous activation (CA) was applied by charging at a constant current rate of 0.1 C during the initial cycle. For the other cell, an intermittent activation (IA) strategy was employed, in which a four-step activation process was designed: Different current rates were applied in different voltage intervals, following the principle that higher voltages correspond to lower current rates. Specifically, charging was conducted at 0.4 C between the open-circuit voltage and 3.32 V, 0.3 C between 3.32 and 3.38 V, 0.2 C between 3.38 and 3.43 V, and 0.1 C between 3.43 and 3.65 V, followed by discharging at 0.1 C.

### DFT Calculations

First-principles density functional theory (DFT) calculations were performed via projector augmented-wave (PAW) and the Perdew–Burke–Ernzerhof (PBE) functions for refinement of the atomic configurations and energy optimization. The energy cutoff point for each calculation was 600 eV, with a difference of no more than 10–6 eV. The system was considered convergent when the atomic force for structural optimization was smaller than 0.015 eV Å^−1^. The data were processed via the spin polarization ordering method.

### COMSOL Simulation

In the application of COMSOL software, for the simulation analysis, the Butler–Volmer equation was used to perform the calculations, through which the electric field distribution within the structure can be obtained. When calculating the concentration distribution within the structure, the Nernst–Planck equation was used as the governing equation, which is given by:

1 $$J_{i} = vc_{i} - D_{i} \nabla c_{i} - z_{i} c_{i} u_{i} E$$

The governing equation and boundary conditions of the electric potential field: − ∇(σ*s*,eff∇ϕ*s*) = *j* *I*,

This condition ensures charge conservation at the reaction interface, all the current flowing from the solid conductive network into the interface must be completely carried away by the ionic current in the electrolyte. This is the core boundary condition for coupling electrode reactions and bulk phase transport. The concentration of lithium ions is jointly controlled by Fick diffusion and ion migration: $\frac{\left(\partial {C}_{{\mathrm{Li}}^{+}}\right)}{\partial t}=-D{\nabla }^{2}{C}_{{\mathrm{Li}}^{+}}$ Electrochemical reaction kinetics and SEI growth boundary conditions, the local current density is determined by the Butler–Volmer equation, and the formula is as follows:

2 $$i_{{{\mathrm{loc}}}} = i_{0} \left[ {\exp \left( {\frac{{\alpha_{a}^{F} }}{RT\eta }} \right) - \exp \left( {\frac{{\alpha_{c}^{F} }}{RT\eta }} \right)} \right]$$

## Results and Discussion

### Comparison of the Electrochemical Properties and In situ Testing

**Fig. 1:**
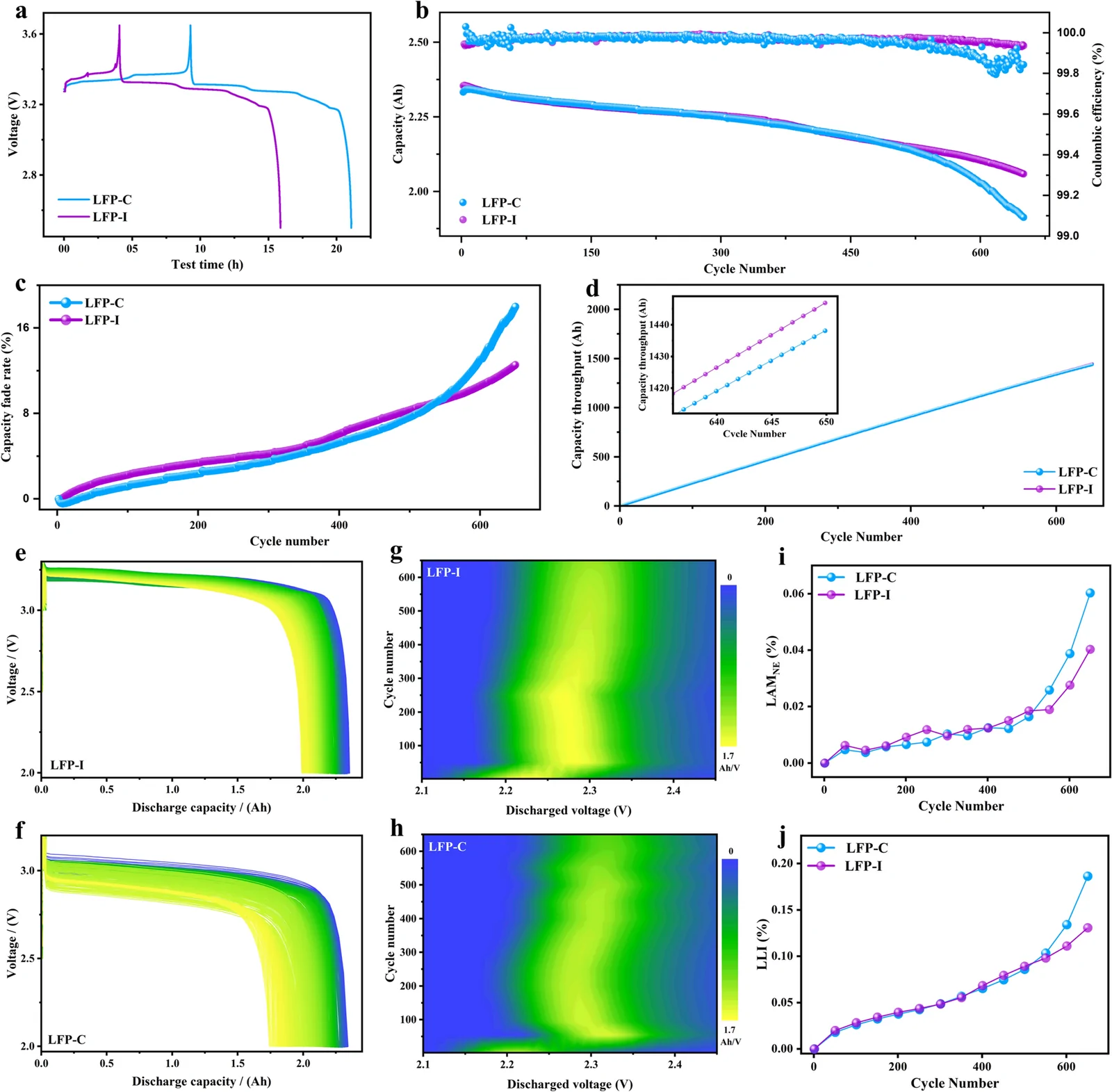
Comparison of the electrochemical properties of pouch-type full cells. **a** Charge/discharge curves at different activations in the first cycle; **b** cycling performance; **c** capacity fade rate; **d** capacity throughput; discharge curves for **e** LFP-I and **f** LFP-C; corresponding differential capacity curves for **g** LFP-I and **h** LFP-C; **i** loss of active material; **j** loss of lithium inventory

Two activation methods were adopted for the pretreatment of the battery. The continuous activation scheme adopted a constant current of 0.1 C charging and discharging, whereas the multi-step activation adopted the strategy of multi-step charging at 0.4, 0.3, 0.2, and 0.1 C; the same 0.1 C was adopted in the discharge stage. Two primary formation protocols, continuous activation with constant current (CA) and multi-step segmented indirect activation (IA), significantly influence the long-term electrochemical performance of LiFePO_4_/graphite batteries. As illustrated in Fig. 1a, the IA protocol applied to the LiFePO_4_ cell (LFP-I) achieves a comparable initial discharge capacity while markedly reducing the formation time, thereby demonstrating enhanced process efficiency. To more clearly differentiate the impact of formation strategies, an accelerated aging protocol—incorporating elevated temperature (45 °C) and over-discharge conditions (2.0 V)—was employed to simulate severe operational environments and expedite degradation processes. Under these stress conditions, after 650 cycles, the LFP-I cell retains 87.45% of its initial capacity, outperforming the conventionally activated counterpart (LFP-C), which retains 82.01%, as shown in Fig. 1b. Moreover, LFP-I has a more stable Coulombic efficiency in the later stage of cycling [14]. Figure 1c shows that during the first 550 cycles, both protocols result in similar capacity fading rates; however, from cycles 550 to 650, the LFP-C degrades rapidly, whereas LFP-I remains relatively stable. In terms of total capacity throughput (Fig. 1d), both exhibit linear growth with cycling, but LFP-I maintains a slight advantage in the later stages. Compared with LFP-I (Fig. 1e), the discharge and charge curves of LFP-C are less overlapping (Fig. 1f). Therefore, LFP-I is more reversible.The corresponding differential capacity curves (dQ/dV in Fig. S1) revealed three discharge peaks (A, B, and C) associated with electrode phase transitions [15].

During the initial 1–50 cycles, peak A in LFP-C shows more pronounced attenuation than that in LFP-I, indicating less effective SEI formation. Figure 1g, h confirms that the LFP-I cell exhibits a more stable dQ/dV profile during both the initial activation phase and prolonged cycling. Further evaluation using the voltage corresponding to thermodynamic equilibrium state (EMF diagnostics) reveals that, under coupled thermal and over-discharge stress conditions, the loss of lithium inventory (LLI) predominates over loss of active material (LAM) as the principal degradation pathway (Fig. 1i, j). When LLI or LAM occurs, characteristic changes appear in the EMF curve. The LFP-I cell displays reduced levels of both LLI and LAM, indicating more stable interfacial chemistry and improved resistance to aging. These findings establish the LFP-I protocol as a more reliable formation strategy for achieving durable battery performance [16, 17].

**Fig. 2:**
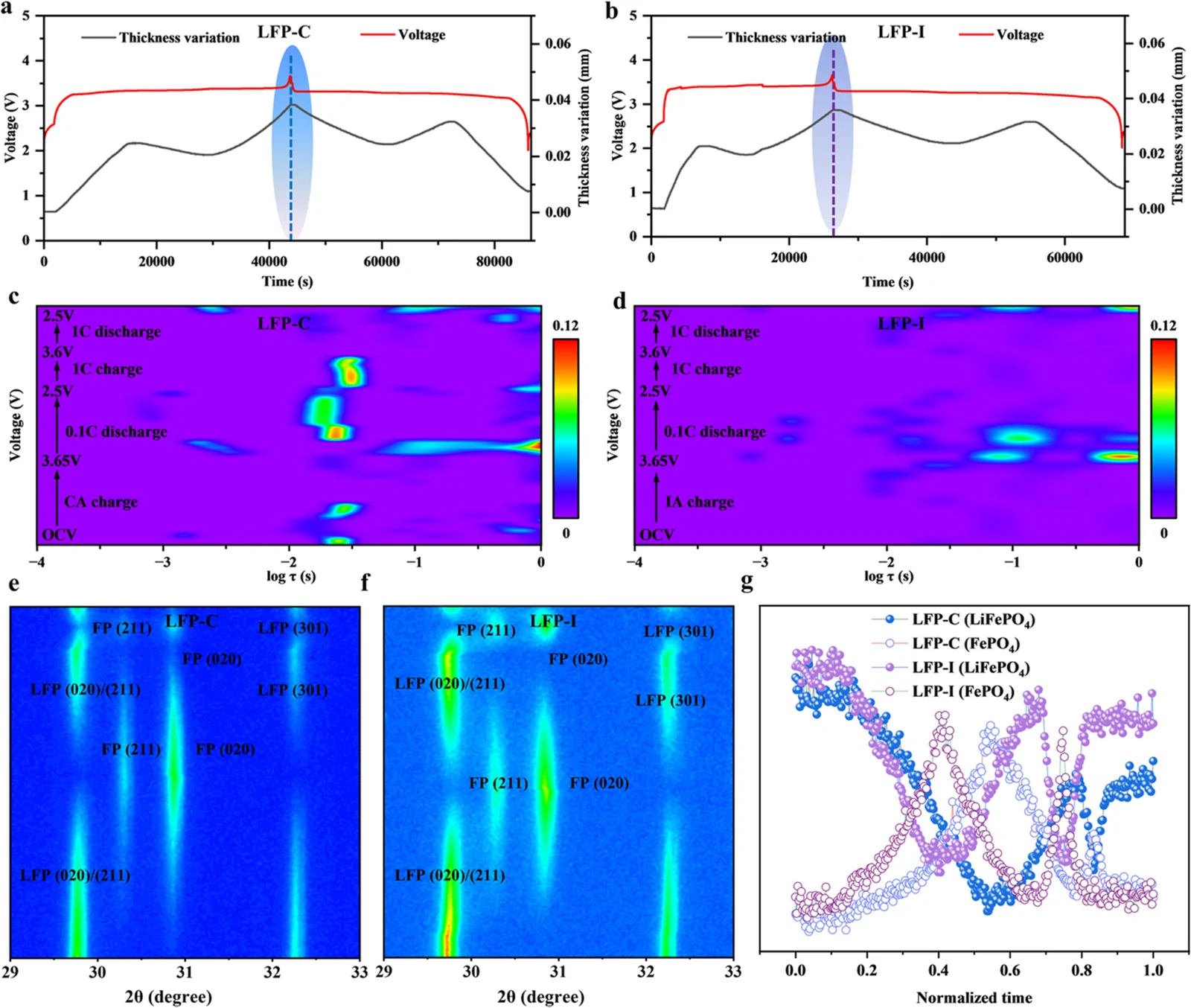
In situ mechanical testing of phase transition and expansion change. In situ expansion analysis for **a** LFP-C and **b** LFP-I; DRT patterns during the initial charge/ discharge process for **c** LFP-C and **d** LFP-I; in situ XRD patterns of the evolution for **e** LFP-C and **f** LFP-I in 2.5–3.65 V; **g** in situ XRD patterns for the distribution of different phases

In situ expansion test (IEST-SWE2100, Initial Energy Science & Technology Co., Ltd., IEST) was conducted to analyze the pressure changes during the charge and discharge process. At the beginning of charging at 0.1C, graphite expansion dominates because LiFePO_4_ contraction is minimal. Subsequently, an increase in voltage leads to volume expansion in the LFP-C, primarily due to Coulombic repulsion along the *c*-axis. At higher states of charge, cathode contraction ceases while graphite expansion continues, producing a renewed thickness increase, with the reverse trend observed during discharge. At the onset of discharge, a reversible expansion occurs, resulting in a characteristic M-shaped expansion curve. This expansion–contraction behavior becomes more pronounced in the LFP-C, indicating that the material undergoes uninhibited dimensional changes during CA process, reflecting limited mechanical reversibility (Fig. 2a). Notably, compared to LFP-C, the LFP-I exhibits significantly smaller pressure variations in the in situ expansion test, suggesting enhanced structural stability. The ordered and thin EEI film on the surface of LFP-I contributes to increase the mechanical strength of the particles (Fig. 2b). The kinetic changes in the complex electrochemical system during the battery aging process were characterized by the distribution of relaxation times (DRT). The peaks at 10^−2^ < *τ* < 10^0^ and 10^−4^ < *τ* < 10^−2^ s represent the charge transfer resistance (*R*_ct_) and Li-ion transport resistance at the electrode–electrolyte interface (*R*_EEI_), respectively [18]. The high peak intensity observed for LFP-C at 0.1 C is primarily attributed to interfacial reconstruction initiated during the early stages of electrochemical reaction. At an elevated rate of 1 C, pronounced differences emerge, with the peak intensities associated with EEI formation and charge transfer significantly increased. This suggests that the formation of the EEI substantially influences the initial charge transfer processes. These changes are indicative of intensified side reactions, leading to increased interfacial impedance and accelerated battery aging, wherein excessive and non-uniform EEI formation contributes to the rapid deterioration of kinetic performance (Fig. 2c). In contrast, the IA strategy employed for LFP-I results in the development of a stable and uniform EEI film, which enhances both interfacial stability and charge transfer efficiency (Fig. 2d). Notably, even at a 1 C rate, the peak intensities associated with EEI formation and charge transfer remain largely unchanged, confirming that the activation process effectively stabilizes the interface and mitigates impedance growth. The DRT analysis reveals that the impedance components of LFP-I remain relatively stable with increasing reaction voltage, further indicating that post-aging impedance growth is primarily attributable to the non-uniform thickening of the EEI layer.

To further elucidate the influence of activation protocols on phase transitions during the electrochemical cycling of LFP, in situ XRD measurements were performed at varying current densities in 2.5–3.65 V. During the charging process, the LFP crystal structure gradually transitions from the orthorhombic phase (LiFePO_4_) to the monoclinic phase (FePO_4_). As lithium is extracted, changes in the Fe–O octahedra are accompanied by contractions in lattice parameters, particularly along the *a*- and *c*-axes. The characteristic diffraction peaks of FePO_4_ and LiFePO_4_ shift symmetrically and in opposite directions during discharge, reflecting reversible phase transformation. However, in the case of LFP-C under CA process, incomplete phase transitions leave portions of unreacted FePO_4_ and LiFePO_4_, as evidenced by overlapping diffraction peaks at 0.1 C. Lithium-ion diffusion facilitates the progressive advance of the LiFePO_4_/FePO_4_ phase boundary, creating distinct two-phase coexistence regions. In LFP-C, this phase boundary exhibits elevated resistance to lithium-ion migration, leading to increased polarization. This situation becomes more obvious at a rate of 1 C (Figs. 2e and S2a). The coexistence of the LiFePO_4_/FePO_4_ phases of LFP-C results in poor reaction kinetics characteristics [19]. This observation further demonstrates that during CA process, unstable EEI films tend to form at the electrolyte–electrode interface, which affects the polarization behavior of the battery. During IA process, the diffraction peak intensities of the (020) and (211) planes in LFP-I progressively weaken until complete disappearance, while the corresponding FePO_4_ diffraction peaks gradually intensify. This indicates the continuous transformation of LiFePO_4_ (LFP) to FePO_4_ (FP), during which the diffraction peak positions shift toward higher angles, with the (311) peak of FePO_4_ progressively, and the orthogonal phase completely transforms into the monoclinic phase [20]. Moreover, the starting point of the phase transition has shifted to a lower charging voltage. In particular, under 1 C conditions, the bimodal coexistence phenomenon of LiFePO_4_/FePO_4_ was significantly inhibited, confirming that LFP-I has strong chemical reversibility (Figs. 2f and S2b). This observation further confirms that the IA process positively influences the stable formation of the EEI. The evolution of diffraction peak intensities at as a function of the SOC further validates the strong chemical reversibility afforded by the IA strategy. During charge–discharge cycles at 0.1 C, both LFP-I and LFP-C exhibit about linear variations in the intensities of the LFP and FP peaks. However, at 1 C, a pronounced lag in the decline of the FP phase is observed in LFP-C at the onset of discharge under low-stress conditions, whereas under high-stress conditions, the FP phase intensity decreases in concert with the SOC. This behavior suggests that, under CA process, phase transitions become heterogeneously distributed within the electrode particles [21] (Fig. 2g). The implementation of IA strategy mitigates this inhomogeneity by facilitating the formation of a stable EEI. This phenomenon underscores the beneficial role of IA strategy in promoting uniform phase transitions within LiFePO_4_ electrodes, thereby contributing to improved electrochemical performance.

To further verify the role of current directionality, we compared LFP-I (charging-only step-down activation with constant discharge) and LFP-II (charging and discharging both step-down activation) with LFP-C (constant-current activation). The results show that the continuous phase transition from LiFePO_4_ to FePO_4_ is smooth and complete in LFP-I, whereas the incomplete phase transformation (evidenced by the residual FP (211) peak) increases polarization and hinders lithium-ion migration in LFP-II (Fig. S3). Meanwhile, LFP-I exhibits stronger particle mechanical strength and greater compressive resistance in situ stress (IEST, SPFT-2000), maintaining more stable CEI/SEI structures over extended cycling. By contrast, LFP-II suffers from repeated high-current discharges to produce inhomogeneous mixed organic–inorganic interphases with higher impedance (Fig. S4). These comparative results confirm that current directionality indeed plays a dominant role in determining the interfacial composition and transport properties of the EEIs.

### Structural Stability of Cathode Electrodes with Cathode–Electrolyte Interphase Films

**Fig. 3:**
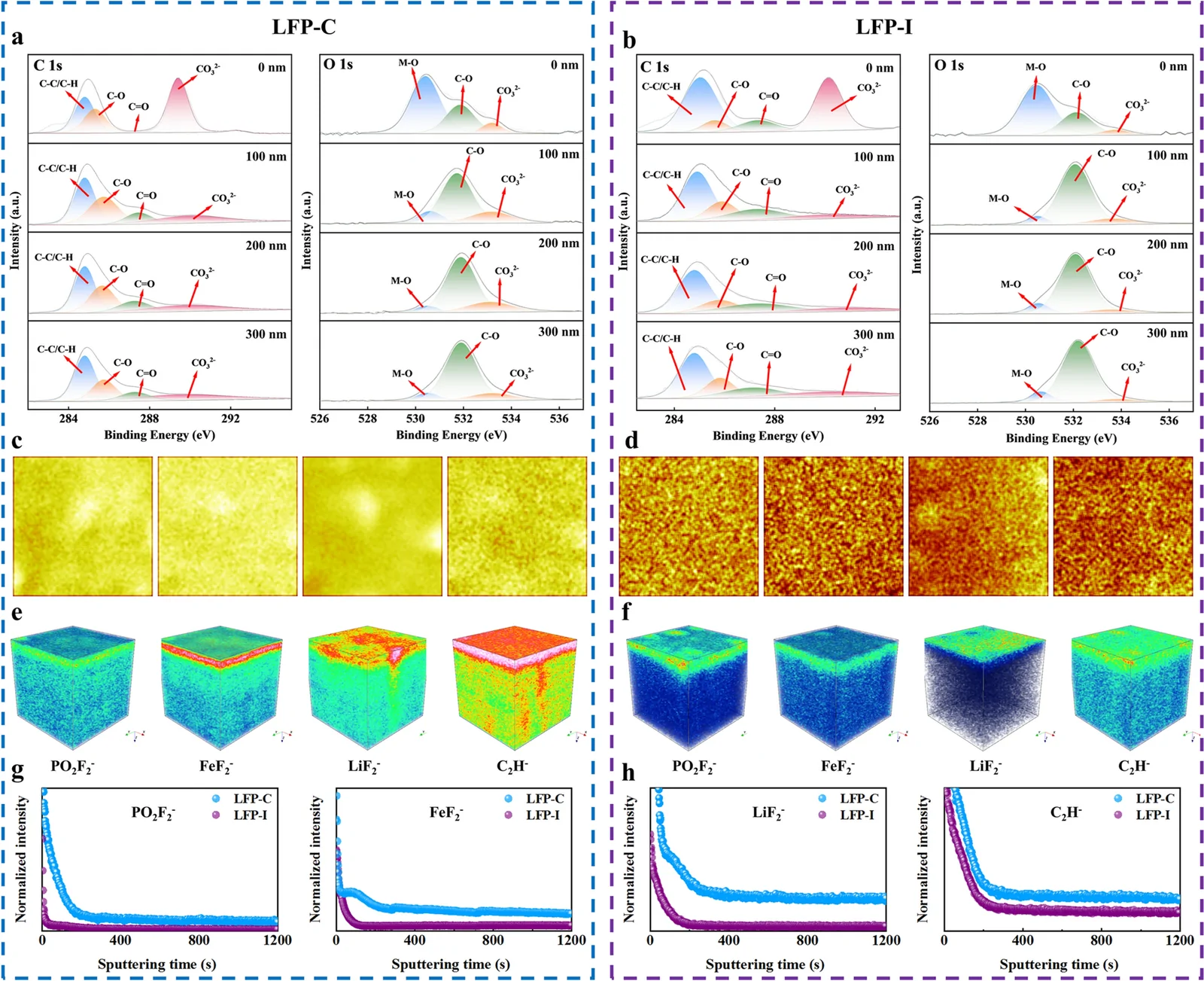
Characterization of depth XPS and TOF-SIMS in pouch-type full cells. 0, 100, 200, and 300 nm etched-depth XPS spectra of C 1*s* and O 1*s* for **a** LFP-C and **b** LFP-I; concentration distributions (PO_2_F_2_^−^, FeF_2_^−^, LiF_2_^−^, and C_2_H^−^) for **c** LFP-C and **d** LFP-I; 3D compositions for **e** LFP-C and **f** LFP-I; corresponding depth profile curves for **g** PO_2_F_2_^−^, FeF_2_^−^ and **h** LiF_2_^−^, C_2_H^−^

XPS was conducted to identify the elemental distribution and chemical valence of the cathode–electrolyte interface (CEI). The characteristic peaks of C 1*s*, O 1*s*, and F 1*s* were inspected in the XPS survey spectra of LFP-C and LFP-I. The C 1*s* spectra predominantly feature C=O, C–O, CO_3_^2−^ and C–C/C–H bonds, and the formation of C=O, C–O, and CO_3_^2−^ bonds is linked to carbonate electrolyte solvent decomposition, whereas the C–C/C–H interactions are related to the binder and conductive material of the electrode [22]. As illustrated in Fig. 3a, compared with LFP-C, the decrease in the peak areas of CO_3_^2−^ in the LFP-I electrode suggests reduced interfacial side reactions. As the etching reached 300 nm, the differences became more obvious, indicating that the crystal structure of LFP-I was well maintained and significantly inhibited the decomposition of the electrolyte on the particle surface. The O 1*s* spectra exhibit peaks for M–O, C–O, and CO_3_^2−^. Remarkably, LFP-I shows a stronger M–O peak than LFP-C, indicating that the dissolution of TM is significantly inhibited (Fig. 3b). Additionally, the decreased CO_3_^2^ peak area in the LFP-I electrode also suggests less electrolyte decomposition. In the F 1*s* spectra, compared with that of the LFP-C electrode (Fig. S5), the intensity of LiF/C-F for LFP-I is markedly reduced from 0 to 300 nm (Fig. S6), indicating enhanced electrolyte stability and improved cycling performance of the cycled electrode material [23]. This suppression effectively mitigates electrolyte decomposition and parasitic side reactions at the electrode–electrolyte interface, thereby extending cycle life and enhancing battery safety. Notably, time-of-flight secondary ion mass spectrometry (TOF-SIMS), integrated with three-dimensional imaging capabilities, enables detailed structural characterization of complex organic species and provides comprehensive visualization of the spatial distribution of chemical components within materials. Analysis reveals that organic fragments (e.g., C_2_H^−^) and phosphorus-containing compounds (e.g., PO_2_F_2_^−^), primarily derived from the decomposition of electrolyte solvents and salts, predominantly accumulate on the outer surface layer of the CEI film [24]. In contrast, characteristic fragments such as FeF_2_^−^ and LiF_2_^−^ typically originate from the decomposition of lithium hexafluorophosphate (LiPF_6_) or from interfacial reactions, indicating their deeper integration into the interphase structure. As a predominant component of the solid–electrolyte interphase (CEI), the presence of fragments suggests electrolyte degradation during lithiation/delithiation. The chemical mapping image of the LFP-C electrolyte (Fig. 3c) reveals that the content of organic species, *P*-compounds, and lithium hexafluorophosphate is significantly greater than that of the LFP-I electrolyte (Figs. 3d and S7 in 3D distribution), confirming that LFP-C suffers from stronger side reactions at the interface. From the 3D distribution and profile depth, compared with those of LFP-C (Fig. 3e), the intensities of PO_2_F_2_^−^ and FeF_2_^−^ in LFP-I clearly decrease as the etching time increases, and the signal decreases to almost zero after 100 s of etching (Fig. 3f). Reactions with organic electrolytes result in surface structural degradation and the formation of transition metal fluorides within the inner layer of the CEI [25]. The CEI formed in the LFP-I electrolyte is considerably thinner. Moreover, in the case of LFP-C, LiF_2_^−^ fragments are concentrated at the surface and persist throughout the entire etching process. In comparison, the LiF_2_^−^ signal intensity in LFP-I is significantly lower, indicating that electrolyte decomposition is effectively suppressed under IA conditions. The sustained presence of C_2_H^−^ signals over sputtering time suggests substantial involvement of electrolyte components in the formation of the cathode–electrolyte interface, with the CEI primarily composed of organic species resulting from carbonate decomposition (Fig. 3g). Depth profiling reveals a sharp decline in the C_2_H^−^ signal intensity for LFP-I after 200 s of sputtering, implying that the IA strategy effectively limits cathode-electrolyte side reactions, facilitates the formation of a thin and uniform CEI composed of small organic molecules, and mitigates the dissolution of transition metal cations (Fig. 3h) [26].

**Fig. 4:**
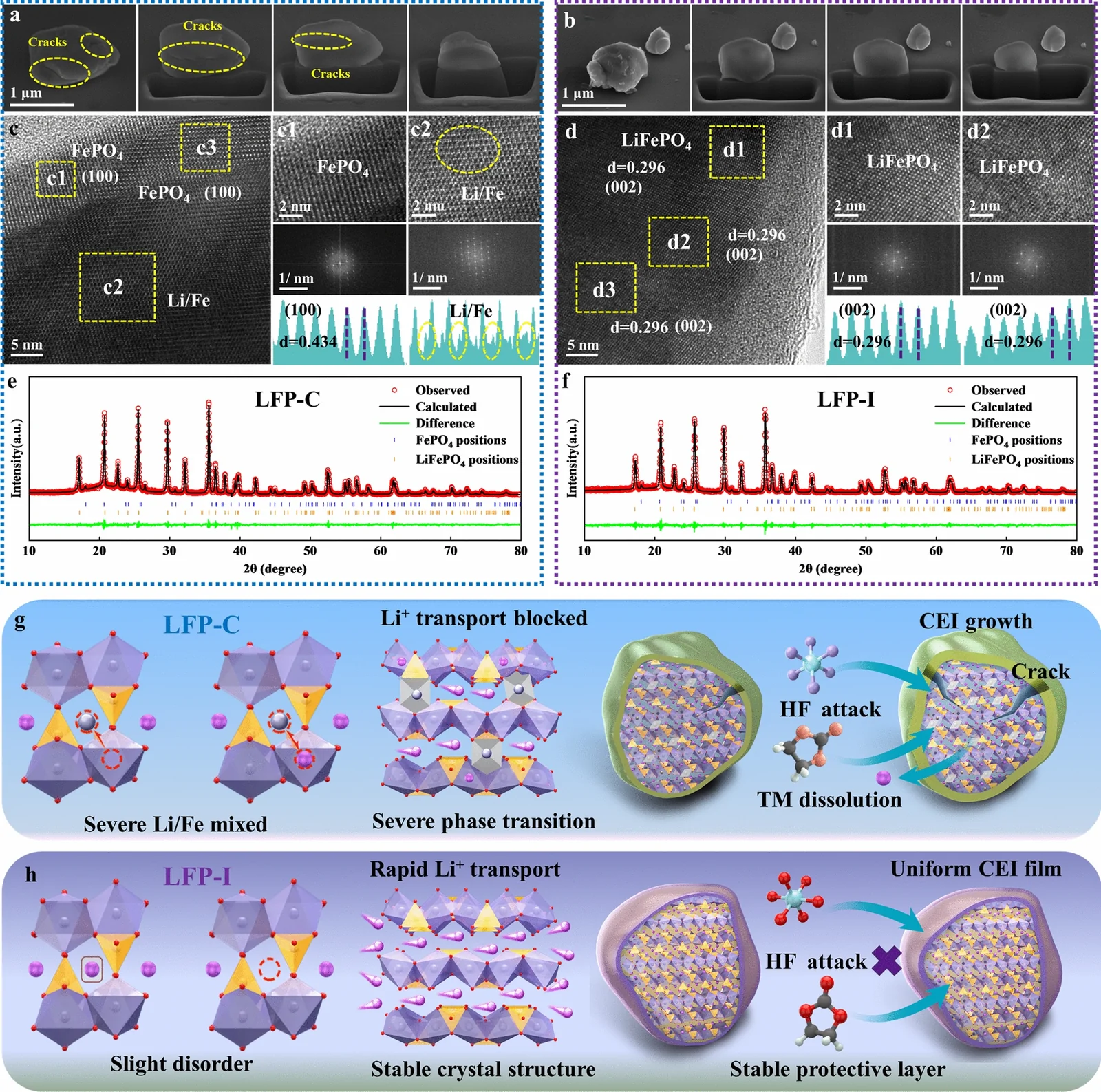
Characterization of the morphology and schematic diagram of the cathode after cycling. FIB-SEM with continuous cutting of the entire single particle for **a** LFP-C and **b** LFP-I; HR-TEM with FFT image of **c** LFP-C and **d** LFP-I; Rietveld refinement results of **e** LFP-C and **f** LFP-I; schematic diagram of **g** LFP-C and **h** LFP-I

The cycled electrodes were analyzed via a focused ion beam (FIB, SCIOS, FEI) with scanning electron microscopy (SEM) and high-resolution transmission electron microscopy (HR-TEM) to investigate their microstructural evolution and phase transformations. During long-term cycling, the LFP-C cathode suffers from severe surface and intragranular microcracks due to stress–strain mismatch and rapid structural degradation induced by side reactions (Fig. 4a). In contrast, the LFP-I cathode displayed nearly no cracks on the particle surface or within the crystalline domains, as the IA strategy promoted the formation of a stable cathode–electrolyte interphase (CEI) layer, which facilitated interfacial energy release and effectively protected the cathode (Fig. 4b). HR-TEM characterization of the cycled cathode demonstrated that LFP-C particles were encapsulated by a thick and heterogeneous CEI layer. Fast Fourier transform (FFT) analysis of region c1 revealed a measured lattice spacing of 0.434 nm, corresponding to the (100) crystal plane of FePO_4_ (FP), and region c3 exhibited the same (100) crystal plane of FP (Fig. S8), whereas FFT analysis of the particle core region (c2) revealed a mixed Li/Fe distribution, demonstrating the coexistence of the FP and LFP phases in this domain [27] (Fig. 4c). These observations are in full agreement with previous results obtained through deep-etching XPS and TOF-SIMS analyses.

These results verify that lithium loss and iron dissolution mainly occur at particle surfaces, which is consistent with the literature. In contrast, the LFP-I surface possesses a thin and uniform CEI layer; region d1 has a lattice spacing of 0.296 nm, corresponding to the (020) plane of LFP, whereas other interior regions (d2 and d3) also display the (020) plane of LFP [28] (Figs. 4d and S9). This phenomenon suggests that IA strategy facilitates the formation of a stable and uniform CEI layer, which promotes homogeneous interfacial energy release and mitigates the development of microcracks within primary particles. The crystal structure was characterized via Rietveld refinement based on XRD analyses conducted on both pristine and cycled LFP samples. Occupancy rates for LFP and FP phases were determined, revealing a substantial presence of the FP phase in the LFP-C sample under CA conditions (Fig. 4e and Table S1). A series of complex phase transitions and parasitic reactions disrupted the crystal lattice, accompanied by significant stress accumulation, which is a primary cause of intragranular crack formation. In contrast, the LFP-I sample under IA strategy exhibited higher LFP phase occupancy and relatively stable local particle environments (Fig. 4f), with the material largely preserving its pre-cycling crystal structure (Fig. S10). The mechanism diagram illustrates the corresponding electrochemical processes at the cathode, where the lithium-ion diffusion coefficient is closely associated with the CEI formed near LFP particle surfaces. In the LFP-C cell, extensive side reactions and HF corrosion give rise to a poorly conductive CEI layer and TM dissolution at the cathode. Anisotropic phase transformation-induced two-phase mismatch strain (Li/Fe disorder), combined with high internal stress during lithiation/delithiation cycles, contributes to interfacial energy relaxation and microcrack propagation [29]. This accumulated stress leads to the shrinkage of FeO_6_ octahedra, a reduced lithium-ion diffusion rate, and decreased conductivity, thereby degrading electrochemical performance (Fig. 4g). Conversely, the IA strategy approach in LFP-I promotes the formation of a structurally stable CEI layer. This CEI not only minimizes oxidation of the LFP cathode by the electrolyte but also suppresses morphological degradation and eliminates structural defects in LFP particles (Fig. 4h). The small volume change in LFP-I enhances the mechanical integrity of LFP through rapid kinetics on the surface during the lithiation/delithiation processes [30].

The activation process affects the contraction and expansion of the lattice during lithiation/delithiation and the redox of Fe in the crystal. Therefore, the chemical valence states of Fe after cycling were studied via X-ray absorption near-edge structure (XANES) spectroscopy. The pre-edge feature of LFP energy is Fe^2+^ at 7112 eV and Fe^3+^ at 7114 eV, which is due to electrons being more strongly bound to the Fe nucleus than to Fe^2+^, resulting in a corresponding transition to higher energy. For a fully charged LFP cathode electrode, the higher the peak of the pre-edge is to the right, the higher the valence state of Fe, and the more Li-ion transfer can be excited during the charging process.

**Fig. 5:**
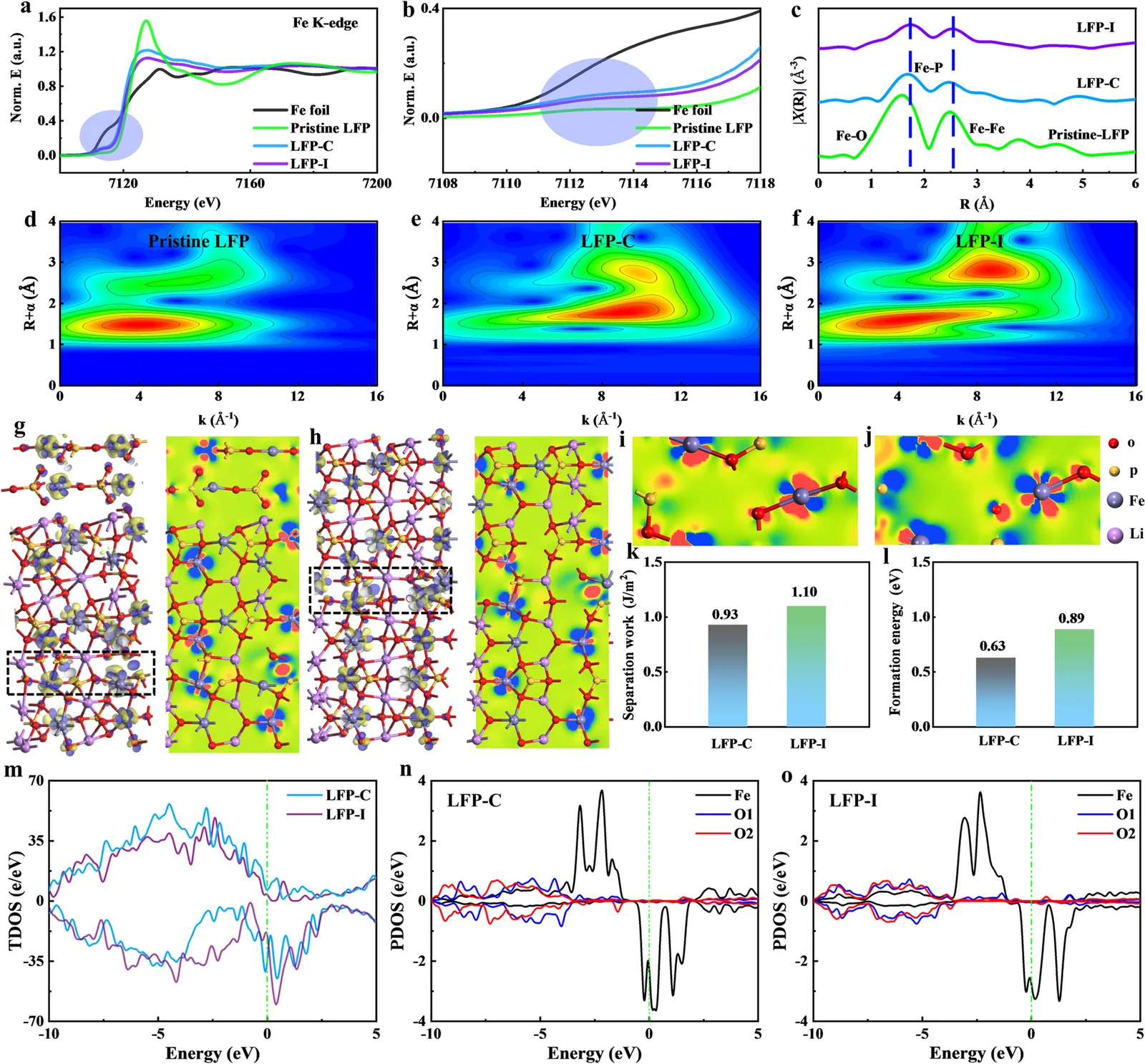
Advanced XANES of the Fe K-edge and density functional theory calculations. **a** XANES spectra of the Fe K-edge before and after cycling; **b** corresponding local magnification; **c** ex situ Fe K-edge EXAFS; 2D Fourier-transformed EXAFS spectra for **d** pristine LFP; **e** LFP-C; **f** LFP-I; 3D and 2D differential charge density distributions of **g** LFP-C and **h** LFP-I; 2D differential charge density distributions of the black box area for **i** LFP-C and **j** LFP-I (corresponds to the LFP-electrolyte interface); **k** interfacial stability of separation work; **l** vacancy formation energy; **m** total and partial density of states plots for **n** LFP-C and **o** LFP-I

As indicated in Fig. 5a, compared with those of pristine LFP, the peak positions and shapes of all the materials changed after cycling, which proved that the material underwent a series of phase transitions during cycling. The olivine structure of LFP inherently possesses excellent stability and electrochemical properties, with its complex electronic structure and coordination environment serving as the foundation for its superior performance. The LFP-I showed a higher Fe^3+^ valence state, indicating that the integrity of the material was well maintained. In contrast, the phase transition is severe, and the peak shift is relatively small for LFP-C (Figs. 5b and S11). A reduction in iron valence or alterations in the crystal structure can result in decreased electrical conductivity and impaired lithium-ion diffusion kinetics, ultimately compromising the rate capability and cycling stability of the battery [31]. To directly probe the local environment of Fe during cycling, extended X-ray absorption fine structure (EXAFS) analysis—highly sensitive to local chemical and structural variations—was employed. In this analysis, oxygen constitutes the first coordination shell around iron, while phosphorus and iron form the second and third shells, respectively. Spectral analysis reveals that the Fe–O and Fe–P peak intensities in pristine LFP are markedly higher than those in the cycled samples, with correspondingly higher coordination numbers for Fe–O and Fe–P bonds. These differences are indicative of changes in the material’s physical and chemical characteristics, including stability, reactivity, and electronic conductivity (Fig. 5c). To further distinguish backscattering atoms and evaluate the local Fe environment, two-dimensional contour Fourier-transformed Fe K-edge EXAFS mapping was conducted. LFP-I exhibits local structural features comparable to this of pristine LFP (Fig. 5d). However, after cycling, compared with LFP-C (Fig. 5e), the Fe–O and Fe–P interatomic distances and peak intensities in LFP-I remain relatively unchanged (Fig. 5f), signifying a stable and reversible local coordination environment.

To assess the effects of different activation processes on the electron transport capacity, density functional theory (DFT) was used to evaluate the structural stability [20]. The corresponding DFT structure was constructed on the basis of the crystal model after cycling (Figs. S12 and S13). The 3D and 2D charge difference isosurfaces of the two systems are 0.07, where blue represents electron density enrichment and yellow represents electron density dissipation. The 3D and 2D charge difference diagrams show that LFP-I presents a wider range of electron density variation areas (Fig. 5h), indicating that the interface electronic interaction is stronger than that of LFP-C (Fig. 5g), which helps improve the interface combination and system stability. The interface area to be compared is shown in the black dotted box. The 2D charge difference screenshots have a unified scale range, which is convenient for comparison and analysis to reflect the difference in electron transfer in different directions. LFP-I has a more significant electronic interaction on the horizontal cross-section of the interface region, which is conducive to the stability of the interface region and the inhibition of Fe dissolution [32] (Fig. 5i, j). As seen from the calculation results in Fig. 5k, the higher the separation work value is, the stronger the interface bonding ability is, and the interface bonding ability of LFP-I is stronger than that of LFP-C overall. This phenomenon is conducive to the overall stability of the system; the more positive the value of the vacancy formation energy is, the more difficult it is to form vacancies. The calculation results in Fig. 5l show that Fe in the interface region of the LFP-I system has a greater vacancy formation energy value, which indicates that the difficulty of Fe ejection from the interface of the LFP-I system is greater than that of the LFP-C system. The results indicate that the LFP-I system exhibits a greater capacity to suppress Fe dissolution compared to the LFP-C system. Analyzing the overall trend of the TDOS curve, the LFP-I system displays a slightly smoother profile than that of LFP-C (Fig. 5m), suggesting improved orbital hybridization and electron delocalization, which contribute to enhanced structural and electronic stability. Additionally, while the spin-up peak values near the Fermi level are comparable between the two systems, the LFP-I system exhibits a notably higher spin-down peak value. This suggests superior electronic properties and implies that LFP-I may possess enhanced electrical conductivity relative to LFP-C. PDOS analysis of Fe and O in the interface region was carried out. Fe-O1 involves bond cooperation parallel to the interface, whereas Fe-O2 involves bond cooperation perpendicular to the interface [33]. Compared with the LFP-C system in the energy range of − 7.5 to − 5 eV, Fe-O1 and Fe-O2 have better peak resonance (peak overlap) phenomena, which means that Fe may have stronger interactions with different O surroundings in the LFP-I system. Additionally, it was observed that the variation trends of the O1 and O2 curves in the LFP-C system differ significantly, indicating that the O atoms surrounding Fe are situated in highly heterogeneous local chemical environments. This heterogeneity adversely affects the overall coordination between Fe and the surrounding oxygen atoms [34] (Fig. 5n, o). In contrast, the analysis suggests that Fe exhibits stronger binding with oxygen in the interfacial region of the LFP-I system, which is more favorable for suppressing Fe dissolution. These findings are consistent with the previously discussed calculations of Fe vacancy formation energy.

### Structural Stability of Anode Electrodes with Solid–Electrolyte Interphase Films

**Fig. 6:**
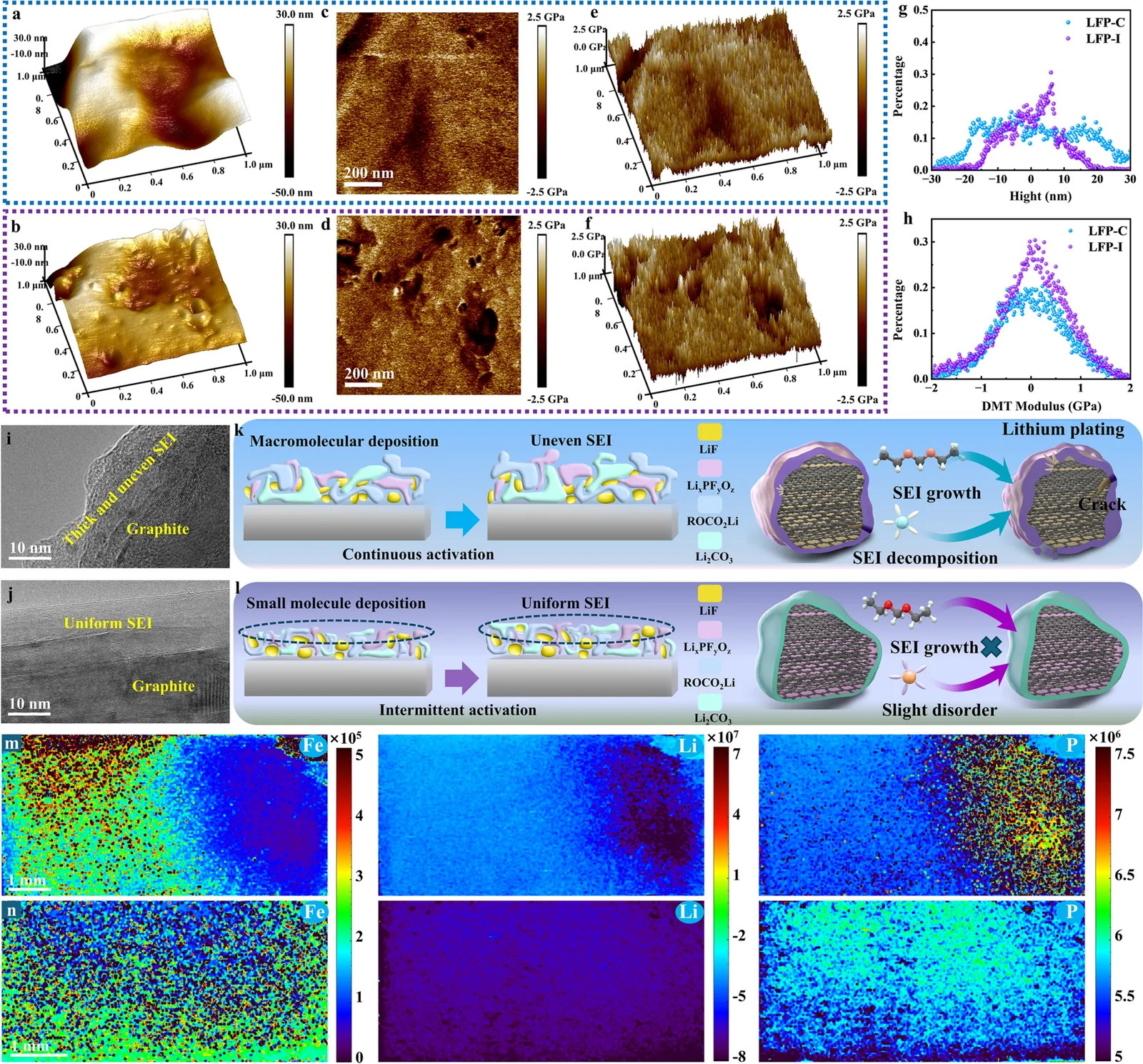
Characterization of the morphology and schematic diagram of the anode after cycling. AFM analysis of 3D height images for **a** LFP-C and** b** LFP-I; 2D Young’s modulus for **c** LFP-C and **d** LFP-I; 3D Young’s modulus for **e** LFP-C and** f** LFP-I; **g** surface height distribution; **h** Young’s modulus histogram; SEM for **i** LFP-C and **j** LFP-I for anode graphite; schematic diagram of **k** LFP-C and **l** LFP-I for anode graphite; LA-ICP-MS with Fe, Li, P for **m** LFP-C and **n** LFP-I for anode graphite

The physicochemical properties of the anode material were examined using atomic force microscopy (AFM) to characterize the morphology of the solid electrolyte interphase (SEI) film after the initial cycle. As shown in Figs. S14 and S15, the 2D images reveal that particles in both LFP-C and LFP-I possess similar submicron-scale structures. However, the 3D images of directly activated LFP-C particles display slightly increased surface roughness, which may contribute to the development of a thicker SEI film and associated modifications in surface morphology (Fig. 6a). In contrast, the LFP-I material demonstrates a denser and more uniform graphite surface, confirming the formation of a more homogeneous SEI film (Fig. 6b). To assess the local mechanical stress of SEI films formed under different activation protocols, AFM modulus mapping was employed alongside corresponding Young’s modulus maps [35]. Given that the outer SEI layer is primarily composed of organic components, LFP-C particles tend to form thicker SEI films, resulting in a lower surface Young’s modulus (Fig. 6c, e). In contrast, the higher Young’s modulus observed in LFP-I particles suggests enhanced resistance to volumetric fluctuations during lithiation and cycling, thereby contributing to improved structural stability of the anode (Fig. 6d, f). Owing to the increased SEI thickness of the outer layer in LFP-C, the surface height distribution histogram confirms the height and corresponding roughness of the non-uniform SEI films (Fig. 6g), and the Young’s modulus histogram further indicates the uniformity of the stress distribution (Fig. 6h).

Moreover, the SEM images show that the SEI film of LFP-I is more uniform and dense (Fig. 6i), the SEI film of LFP-C is thicker and rougher for anode graphite, and this result corresponds to the AFM results (Fig. 6j). The mechanism schematic visually illustrates the corresponding SEI formation process, demonstrating that the SEI thickness is strongly correlated with the activation procedure [36]. With the morphological rearrangement of the electrolyte during the cycling process, the accumulation of byproducts from the decomposition of the electrolyte increases the SEI thickness, where CA process leads to increased SEI thickness. The thick and non-uniform SEI layer impedes ion transport, deteriorates ionic kinetics, and consequently induces irreversible LixC_6_ formation and anode material degradation (Fig. 6k). During IA process, the increased reaction current promotes the deposition of more small molecules on the anode side, while these corresponding small molecules form a thin and uniform SEI film that suppresses the dissolution of cathode TMs at the anode (Fig. 6l). The IA process simultaneously forms uniform CEI and SEI films on both the cathode and anode, confirming the enhancement effect of the EEI on the electrochemical performance. To explore the dissolution of metal elements at the anode electrode, trace element analysis was carried out via laser ablation inductively coupled plasma-mass spectrometry (LA-ICP-MS). The dissolved Fe at the cathode electrode is transported through the electrolyte to the anode electrode and deposited on the anode electrode side. There is a greater signal distribution of Fe with an uneven content on the anode electrode side of LFP-C, which proves the dissolution of more Fe and the existence of an uneven SEI film (Fig. 6m). In contrast, the content of Fe on LFP-I is extremely low, and the strength distribution is uniform, verifying the composition of a uniform SEI (Fig. 6n). Moreover, the distribution of Li and P further verified that IA strategy could form a stable SEI film while modifying both the cathode and anode electrode sides to prevent the electrolyte from damaging the electrode. Meanwhile, ICP-OES measurements demonstrate a significant suppression of Fe dissolution in the LFP-I (Fig. S16). Theoretical calculations showed that the lower adsorption energy in LFP-I facilitates lithium-ion migration and contributes to improved electrochemical stability. This further proves that Fe dissolves on the cathode electrode side and is difficult to detach from the anode electrode for LFP-C. This is obviously not conducive to electron conduction and causes the electrode to damage (Figs. S17 and S18).

Finite element analysis was performed via COMSOL software to simulate the mechanism of the SEI film under electric fields under two different activation conditions in anode electrodes for graphite. Current density is a critical parameter influencing the formation of the CEI film. During the charging process, the spatial electric field distribution on the LFP-C surface exhibits a pronounced intensity gradient, resulting in the development of irregular microscopic SEI protrusions on the anode surface. As electrochemical cycling progresses, the roughness of the SEI film increases, and the electric field becomes increasingly concentrated at the tips of these protrusions [37].

**Fig. 7:**
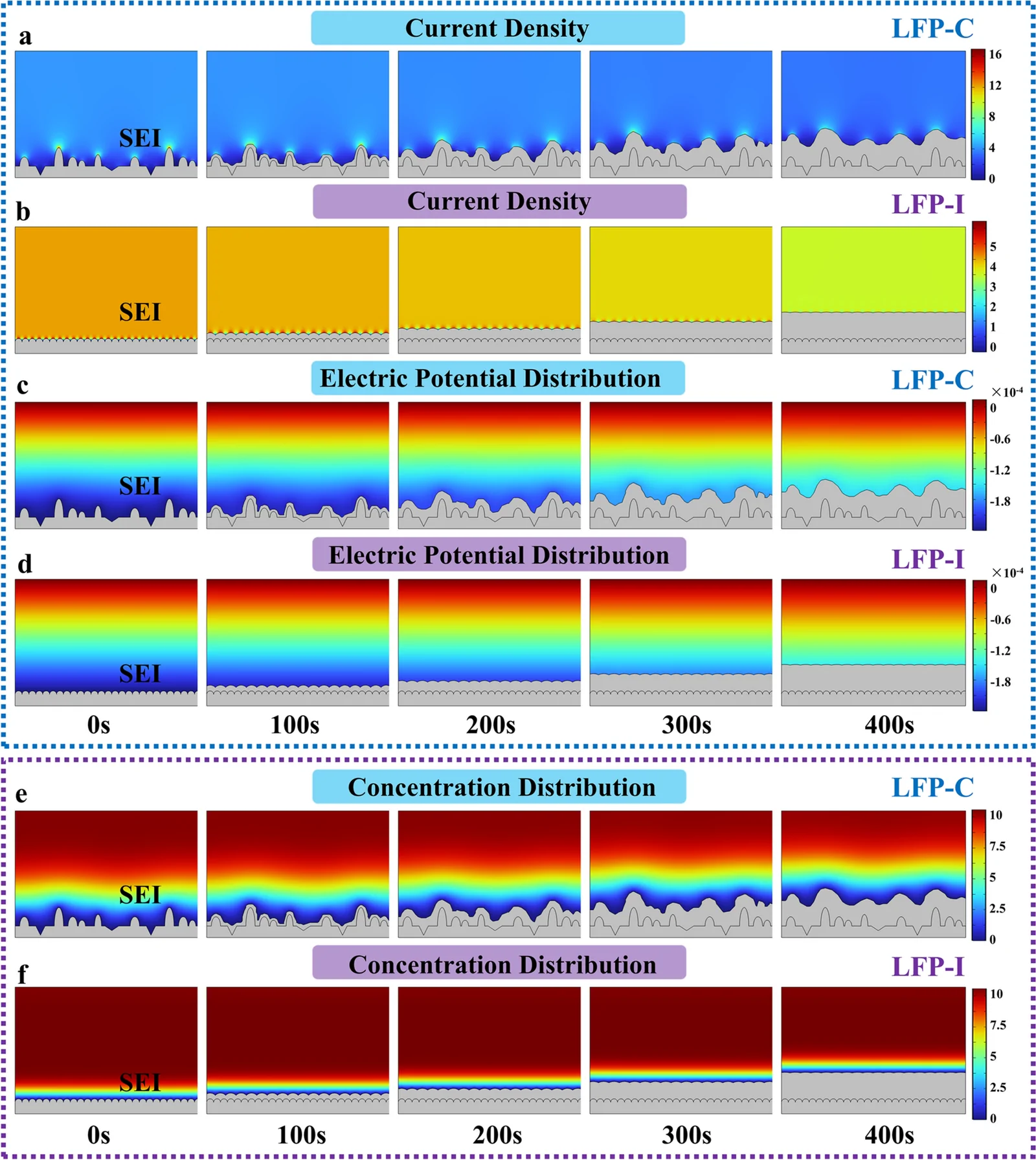
Simulation diagram of the electric field with COMSOL software in anode electrodes for graphite after 700 cycles. (The gray part represents the growth process of different SEI films.) Current densities of **a** LFP-C and **b** LFP-I; electric potential distributions of **c** LFP-C and **d** LFP-I; and Li^+^ concentration distributions of **e** LFP-C and **f** LFP-I

This localized electric field induces excessive charge accumulation at the protruding SEI features, thereby promoting further growth of the uneven SEI layer (Fig. 7a). In contrast, under IA conditions, small-molecule decomposition products from the electrolyte preferentially adsorb onto the particle surface, enhancing the uniformity of current density distribution across the SEI interface. This leads to a more homogeneous electric field and facilitates uniform Li^+^ diffusion. As a result, the IA strategy effectively regulates charge distribution at the anode interface, promoting the formation of a smooth and compact SEI layer over extended reaction times [38] (Fig. 7b). Concurrently, the uniform electric field and homogeneous lithium-ion flux contribute to the formation of a dense and uniform SEI film. From the perspective of electrochemical potential, comparative analysis with LFP-C indicates that the SEI film formed under the IA protocol can significantly reduce the energy barrier at the electrode interface through enhanced interfacial interactions with the organic layer (Fig. 7c). Following IA strategy, the LFP-I system facilitates reduced electron transfer energy from the high-potential electrode to the low-potential organic layer, a phenomenon consistent with the observed electric field distribution and current density characteristics (Fig. 7d). Moreover, in the anode SEI system, LFP-C has a higher Li^+^ concentration in the bulk phase than at the surface, creating a pronounced Li^+^ concentration gradient. This evidence indicates that severe phase transformation occurred at the LFP-C particle surfaces. The formation of a thick SEI leads to an increased average electrode thickness relative to LFP before cycling (Fig. 7e). The less pronounced Li^+^ concentration gradient observed in the LFP-I system indicates relatively weak concentration polarization effects within the electrochemical environment (Fig. 7f). The more uniform cross-sectional distribution of Li^+^ in LFP-I facilitates consistent and rapid Li^+^ diffusion under identical operating conditions.

### Simulation Diagram with COMSOL Software in Pouch-Type Full Cells

**Fig. 8:**
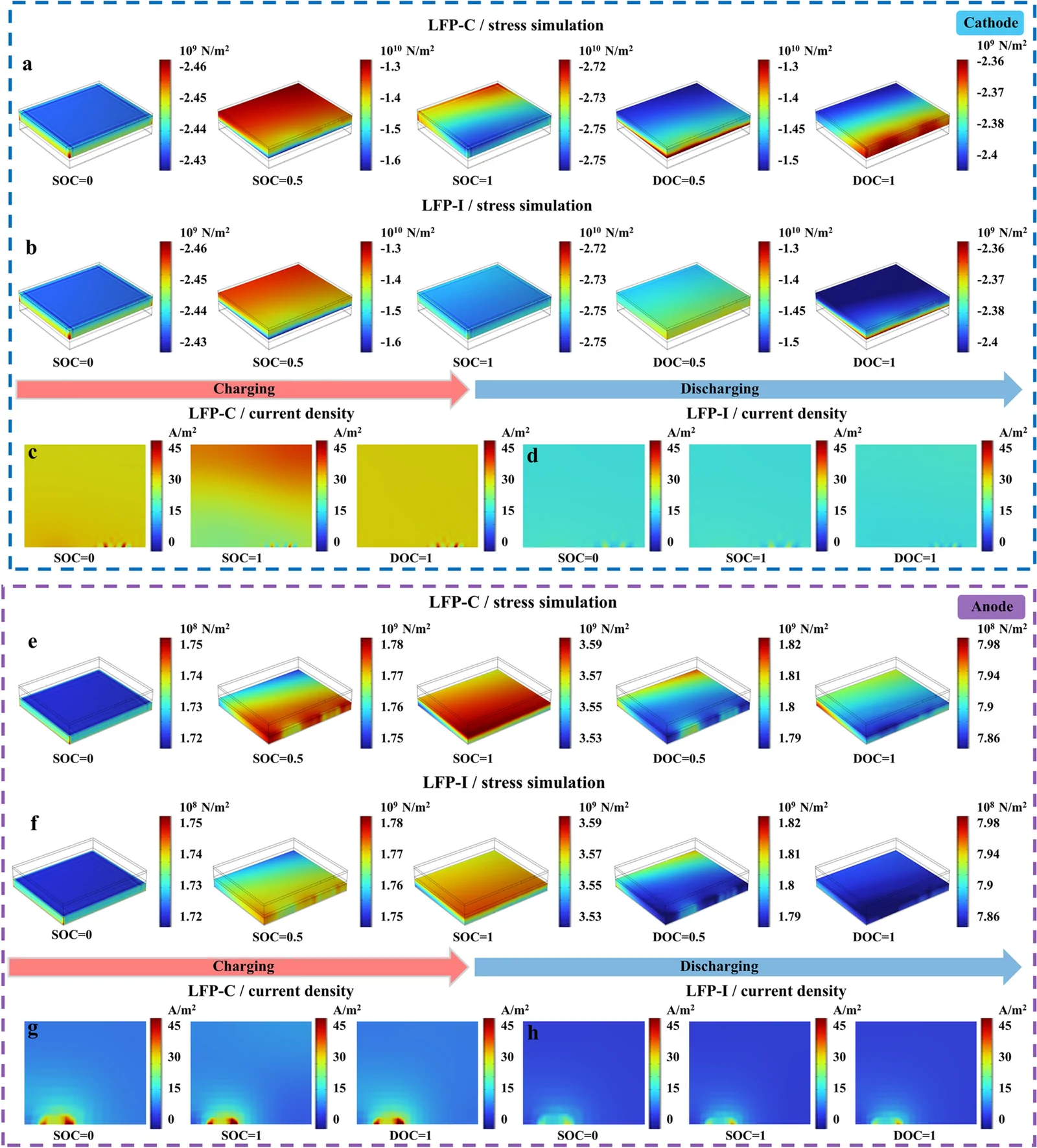
Simulation diagram of stress simulation and current density with COMSOL software in pouch-type full cells after 700 cycles. The stress simulation diagram of **a** LFP-C and **b** LFP-I; current density of **c** LFP-C and **d** LFP-I for the cathode; the stress simulation diagram of **e** LFP-C and **f** LFP-I; current density of **g** LFP-C and **h** LFP-I for the anode

The stress simulation diagram and current density of the LFP pouch-type full cells during the lithiation/delithiation processes were determined via COMSOL software to demonstrate the transport characteristics of LFP-C and LFP-I [39]. The phase transition of the cathode electrode will increase the CEI and generate mechanical stress. Owing to the formation of a thick and uneven CEI and difficult Li-ion deintercalation with a poor Li-ion diffusion coefficient after long-term cycles for LFP-C, the phase transition leads to a corresponding strain difference, which presents stress characteristics. As lithiation/delithiation progresses, the equivalent stress changes continuously; such large and non-uniform stresses are enough to cause unfavorable and irreversible structural damage to the LFP-C electrode (Figs. 8a and S19).

In contrast, LFP-I exhibited a more moderate change in stress concentration and a larger Young’s modulus with a uniform CEI, resulting in a smaller strain value and higher reversibility with a relatively high diffusion coefficient, which improved electrode stress simultaneously after cycling [40] (Figs. 8b and S20). The stress of LFP-I hardly induces severe damage to the structure, which is completely different from that of the LFP-C sample. Li^+^ ions are constantly diffused inside and on the surface of particles, and the concentration of Li^+^ ions dynamically changes. The thickness of the CEI layer directly influences Li^+^ transport and current density distribution in pouch-type full cells. Compared to LFP-C (Figs. 8c and S21), the LFP-I system exhibits a significantly more uniform current density, indicating faster Li^+^ diffusion under identical conditions and reduced phase transition activity (Figs. 8d and S22). These findings confirm that the IA strategy effectively suppresses phase transitions and the uncontrolled growth of the CEI film, thereby optimizing mechanical stress distribution and enhancing Li^+^ transport efficiency.

Degradation on the anode electrode side is also an important indicator of battery aging. To explore the mechanism of the SEI film in battery aging more intuitively, a simulation diagram of the internal stress generation and current density of the anode electrode in the process of lithiation/delithiation is generated. The mechanical stability of the SEI film is directly correlated with its intrinsic stress and Young’s modulus. A higher Young’s modulus indicates greater resistance to deformation with a more uniform and stable SEI film, which gives LFP crystals stronger chemical bonds and a more stable structure. The macromolecular SEI film formed under CA process results in a reduced Young’s modulus, leading to uneven stress distribution across the electrode surface. This accumulated stress propagates from the particle surfaces into the crystal interior, inducing intragranular cracks that permit electrolyte infiltration, thereby accelerating battery performance degradation over time (Figs. 8e and S23). In comparison, LFP-I exhibits a more moderate response, characterized by a higher Young’s modulus and reduced strain under stress concentration. The improved strain homogenization enhances structural reversibility, thereby contributing to superior structural integrity and extended cycle life [41] (Figs. 8f and S24). Moreover, the impact of the CA process is primarily reflected in the electrochemical performance. Compared to LFP-C electrodes (Figs. 8g and S25), LFP-I demonstrates a more uniform current density distribution, indicative of stabilized electrochemical reactions and enhanced electrode kinetics—attributes consistent with the intrinsically stable electrochemical behavior of LFP materials (Figs. 8g and S26). The verification of grid independence demonstrates that the chosen mesh density is sufficient for accurate simulations and the influence of grid size on the final results can be safely neglected (Fig. S27). Full-cell simulations further underscore the critical role of stable EEI films in battery performance. In addressing battery aging, it is imperative to concurrently investigate the mechanisms governing both CEI and SEI formation, as identifying the respective aging pathways in these interphases is essential for the development of structurally stable and high-performance LFP-based systems.

## Conclusions

In summary, the aging mechanism and evolution mode of LiFePO_4_/graphite batteries during long-term cycling at different activations were quantitatively analyzed via TOF-SIMS, FIB, LA-ICP-MS and XANES techniques. Moreover, a combination of DFT calculations and COMSOL simulations of SEI formation and cathode and anode formation in pouch-type full cells further revealed the mechanism of action of the EEI. Under CA process at relatively low current densities, the failure of the cathode electrode CEI and the dissolution of Fe synergistically accelerate the aging of the battery through interfacial side reactions, catalytic decomposition, impedance increases, etc. The SEI film is predominantly composed of inorganic components such as lithium carbonate (Li_2_CO_3_), whereas the anode side tends to form macromolecular and non-uniform SEI films. Under IA strategy across varying current densities, a thin and uniform CEI layer was formed on the LiFePO_4_ cathode, effectively suppressing Fe dissolution and inhibiting the migration of transition metal species. Notably, the generation of organic components such as lithium alkyl carbonates (ROCO_2_Li) was significantly restrained. As a result, the SEI film was composed predominantly of a homogeneous mixture of small organic and inorganic molecules, yielding a thin and uniform interphase. This structural configuration enhances electron transfer kinetics, thereby reducing interfacial resistance and improving the cycling stability of the battery system. Moreover, the stable EEI formed through IA strategy contributes to the stabilization of the crystal structure and mitigates internal stress concentrations arising from repeated phase transformation processes. This, in turn, reduces the dissolution of transition metal cations. Importantly, this IA strategy offers new insights into the aging mechanisms of cathode materials, particularly through an integrated investigation of SEI, CEI, and transition metal dissolution phenomena.

## Supplementary Information

Below is the link to the electronic supplementary material.

Supplementary file1 (DOCX 11152 kb)
