# Supplementary material for: Interfacial Evolution and Accelerated Aging Mechanism for LiFePO4/Graphite Pouch Batteries Under Multi-Step Indirect Activation
Source: Nanomicro Lett. 2026 Jan 5;18:136. doi: 10.1007/s40820-025-01971-2 (PMC12765781; doi:10.1007/s40820-025-01971-2)
Supplement: Supplementary file 1 — Supplementary file1 (DOCX 11152 kb) [file 40820_2025_1971_MOESM1_ESM.docx]

Supporting Information for

**Interfacial Evolution and Accelerated Aging Mechanism for LiFePO_4_/Graphite Pouch Batteries under Multi-Step Indirect Activation**

Yun Liu^1,2^, Jinyang Dong^1,2^*, Jialong Zhou^1,2^, Yibiao Guan^3^, Yimin Wei^4^, Jiayu Zhao^1,2^, Jinding Liang^4^, Xixiu Shi^4^, Kang Yan^2^, Yun Lu^1,2^, Ning Li^1,2^, Yuefeng Su^1,2^*, Feng Wu^2^, Lai Chen^1,2^*

^1^ School of Materials Science and Engineering, Beijing Key Laboratory of Environmental Science and Engineering, Beijing Institute of Technology, Beijing 100081, P. R. China

^2^ Chongqing Innovation Center, Beijing Institute of Technology, Chongqing 401120, P. R. China

^3^ China Electric Power Research Institute, Haidian District, Beijing 100192, P. R. China

^4^ 21C Innovation Laboratory, Contemporary Amperex Technology Co., Ningde 352100, P. R. China

*Corresponding authors. E-mail: [jydong@bit.edu.cn](mailto:jydong@bit.edu.cn) (Jinyang Dong); [suyuefeng@bit.edu.cn](mailto:suyuefeng@bit.edu.cn) (Yuefeng Su); [chenlai144@sina.com](mailto:chenlai144@sina.com) (Lai Chen)

**S1 Experimental Section**

**S1.1 Materials Characterizations**

The crystal structure data of all the samples were examined via Rint-2000 X-ray power diffraction within 10°–80° at a scan rate of 10° min^–1^ (the Rietveld reﬁnement program at 2° min^–1^ by the General Structure Analysis System, GASA). By in situ X-ray diffraction test, a custom-made Swagelok battery with an X-ray transparent aluminum window was used to describe the cell mechanism. To analyze the morphology and microstructure of the samples, scanning electron microscopy (SEM; JEOL, JSM 6400) with a focused ion beam (FIB, FEI, SCIOS) were used, transmission electron microscopy (TEM; JEOL, 2100F) and X-ray photoelectron spectroscopy (XPS, Thermo Fisher ESCALAB 250Xi), TOF-SIMS (ION-TOF GmbH TOF SIMS) and X-ray absorption spectroscopy (XAS) were performed on beamline BL01C1 in the NSRRC to characterize the elemental composition, elemental valence state and corresponding material structure at the surface and depth positions. Laser ablation inductively coupled plasma-mass spectrometry (LA-ICP-MS) was used to characterize the distributions of the corresponding elements. Swelling was monitored using an in-situ swelling analyzer (IEST-SWE2100, Initial Energy Science & Technology Co., Ltd., IEST) with sub-micron resolution, under controlled conditions of 25 ± 1 °C in a thermostatic chamber to avoid thermal interference.

**S1.2 Electrochemical Tests**

To investigate the impact of activation strategies on battery performance, LiFePO_4_/graphite pouch-type lithium-ion batteries with a nominal capacity of 2 Ah were employed in this study. Each cell consisted of a double-sided graphite anode and a double-sided LiFePO_4_ cathode assembled using a winding process. The electrode dimensions were 93.0 × 646.0 mm for the anode and 87.0 × 520.0 mm for the cathode, with corresponding thicknesses of 0.0631 mm and 0.07926 mm, respectively. The N/P ratio of the cells was approximately 1.1. For one cell, continuous activation (CA) was applied by charging at a constant current rate of 0.1 C during the initial cycle. For the other cell, an intermittent activation (IA) strategy was employed, in which a four-step activation process was designed: different current rates were applied in different voltage intervals, following the principle that higher voltages correspond to lower current rates. Specifically, charging was conducted at 0.4 C between the open-circuit voltage and 3.32 V, 0.3 C between 3.32 V and 3.38 V, 0.2 C between 3.38 V and 3.43 V, and 0.1 C between 3.43 V and 3.65 V, followed by discharging at 0.1 C.

**S1.3 DFT Calculations**

First-principles density functional theory (DFT) calculations were performed via projector augmented-wave (PAW) and the Perdew-Burke-Ernzerhof (PBE) functions for refinement of the atomic configurations and energy optimization. The energy cutoff point for each calculation was 600 eV, with a difference of no more than 10–6 eV. The system was considered convergent when the atomic force for structural optimization was smaller than 0.015 eV/Å. The data were processed via the spin-polarization ordering method. The authors extend their gratitude to Xingpeng Shuai from Scientific Compass (www.shiyanjia.com) for providing invaluable assistance with DFT calculations。

**S1.4 COMSOL Simulation**

In the application of COMSOL software, for the simulation analysis, the Butler–Volmer equation was used to perform the calculations, through which the electric-field distribution within the structure can be obtained. As the overpotential increases, the local current density exhibits an exponential rise. Therefore, increasing the overpotential by a given amount can cause the local current density to increase by orders of magnitude, thereby significantly accelerating the rate of the electrochemical deposition reaction. When calculating the concentration distribution within the structure, the Nernst–Planck equation was used as the governing equation, which is given by:

$$J_{i}\text{=}vc_{i}-D_{i}\nabla c_{i}-z_{i}c_{i}u_{i}E$$

Here, J_i_ represents the total flux of the i ionic species; v is the velocity of the liquid at the electrode surface; ci is the concentration of the i ion; D_i_ is the diffusion coefficient of the i ion; is the concentration gradient of the i ion; u_i_ denotes the mobility of the i ion; E is the electric-field strength; and zi is the charge number of the i ion. For cations and anions, the migration flux term carries a positive or negative sign, respectively, reflecting the influence of their opposite charges.

The governing equation and boundary conditions of the electric potential field : −∇(σs,eff∇ϕs) = j I, This condition ensures charge conservation at the reaction interface, all the current flowing from the solid conductive network into the interface must be completely carried away by the ionic current in the electrolyte. This is the core boundary condition for coupling electrode reactions and bulk phase transport. The concentration of lithium ions is jointly controlled by Fick diffusion and ion migration: $\frac{\left( \partial C_{{Li}^{+}} \right)}{\partial t}=-D\nabla^{2}C_{{Li}^{+}}$ Electrochemical reaction kinetics and SEI growth boundary conditions, the local current density is determined by the Butler-Volmer equation, and the formula is as follows:

$$i_{loc}=i_{0}\left[ \exp\left( \frac{\alpha_{a}^{F}}{RT\eta} \right)-\exp\left( \frac{\alpha_{c}^{F}}{RT\eta} \right) \right]$$

The relative Permittivity of LFP is εᵣ=12, the lithium-ion diffusion coefficient is 10⁻^13^ cm²/s. Poisson's ratio is usually lower, approximately ν=0.25, Elastic modulus (Young's modulus) is E = 120 GPa, Electronic conductivity is 10⁻^8^ with exchange current density of 0.1 A/m², and the Phase boundary energy is 0.1-0.5 J/m². When simulating the growth process of the SEI film, The relative Permittivity o is εᵣ=5.

**S1.5 Swelling Tests**

Swelling was monitored using an in-situ swelling analyzer (IEST-SWE2100, Initial Energy Science & Technology Co., Ltd., IEST) with sub-micron resolution, under controlled conditions of 25 ± 1 °C in a thermostatic chamber to avoid thermal interference. The subsequent experiments were performed under constant-current activation (CA) and multi-step segmented indirect activation (IA) protocols, with each test repeated two times to ensure reproducibility. To enable comparison across formats, swelling was reported as the relative thickness change rate (Δt/t_0_ × 100%), which provides a normalized descriptor independent of the absolute cell dimensions. The expansion behavior originates mainly from the graphite anode, which undergoes ~10% lattice swelling upon full lithiation, while the LiFePO_4_ cathode contributes a smaller but non-negligible ~7% volume change during the LiFePO_4_ ↔ FePO_4_ two-phase transition.

**S1.6 In-situ Stress Tests**

The SPFT-2000 device consists of an optical microscope, a pressure measurement system, a displacement measurement system, and a force-displacement control system. Through high-precision displacement and pressure control, it can collect the pressure-displacement curve after the indenter (with a planar size of about 50 μm, customizable) is loaded onto a single particle, and analyze the crushing force of the particle from the sudden change point of the curve. During the testing process, an optical microscope can be used to observe the morphology of the particles before and after pressing, as well as to test the size information of the particles, etc.

**S2 Supplementary Figures**


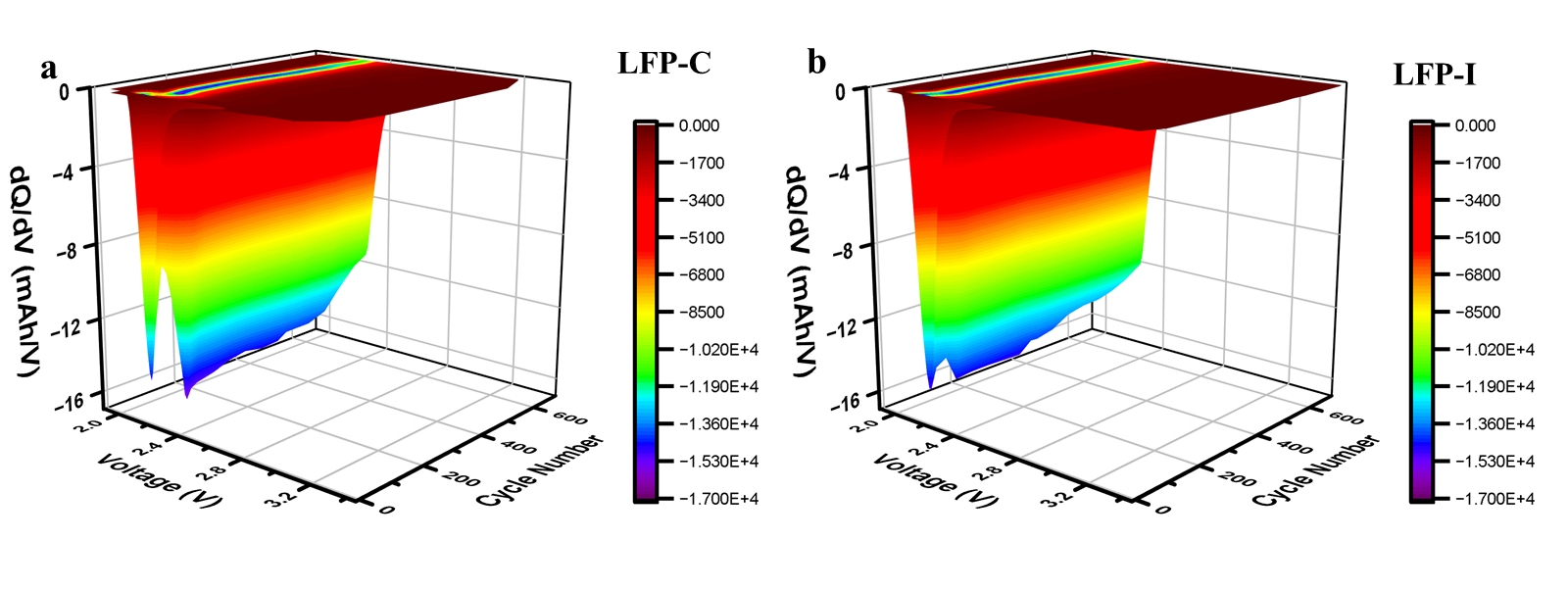


**Fig. S1** The corresponding differential capacity curves of (**a**) LFP-C and (**b**) LFP-I


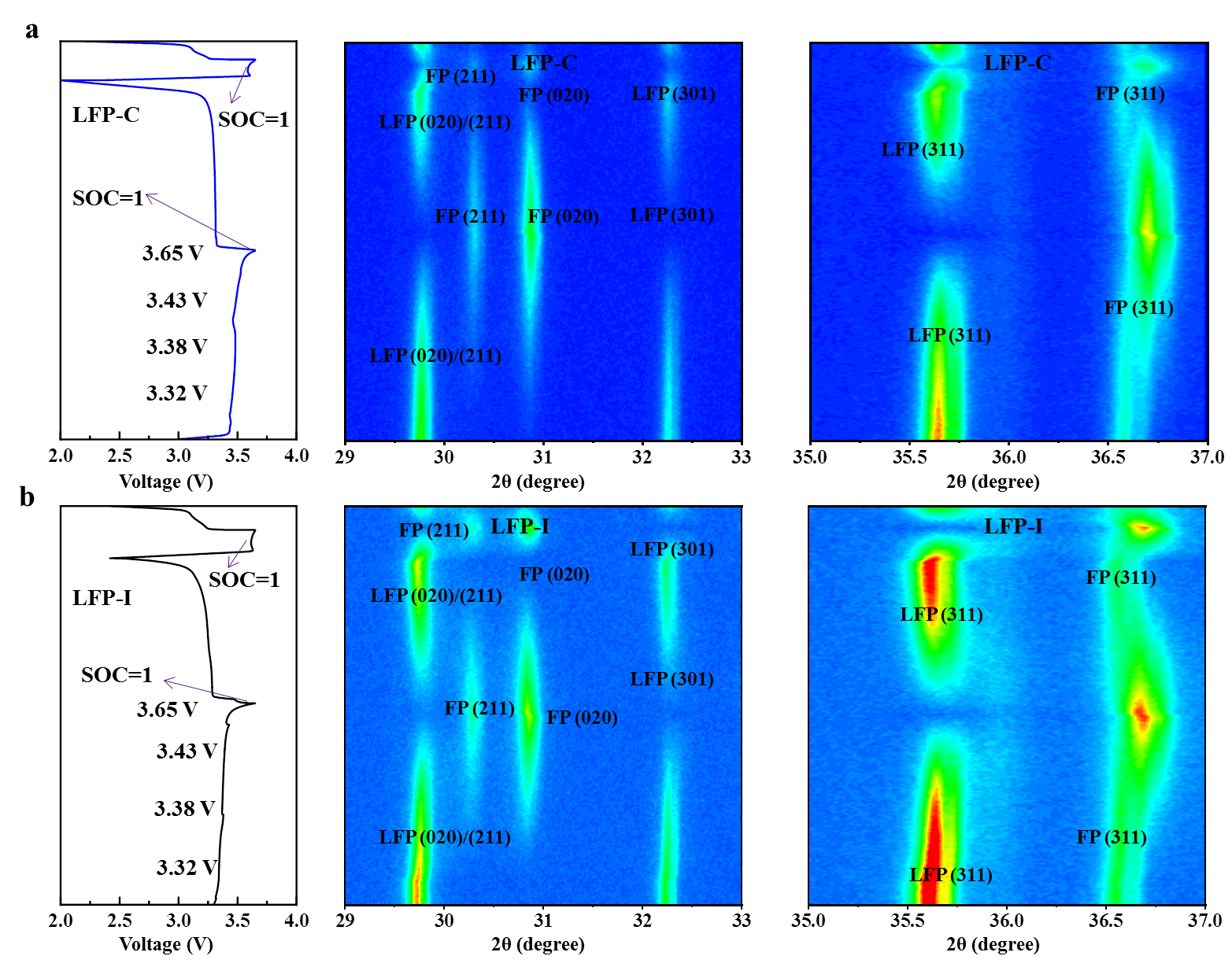


**Fig. S2** Selected contour plots in situ XRD of (**a**) LFP-C and (**b**) LFP-I in 2.5–3.65 V

The applied voltage window for the half-cell tests was 2.5–3.65 V, which corresponds to the conventional charge–discharge range of LFP. Within this range, the reversible phase transitions between LiFePO_4_ (LFP) and FePO_4_ (FP) can be effectively monitored. Both CA and IA formation protocols were implemented under this window to track and compare the structural evolution pathways in real time.


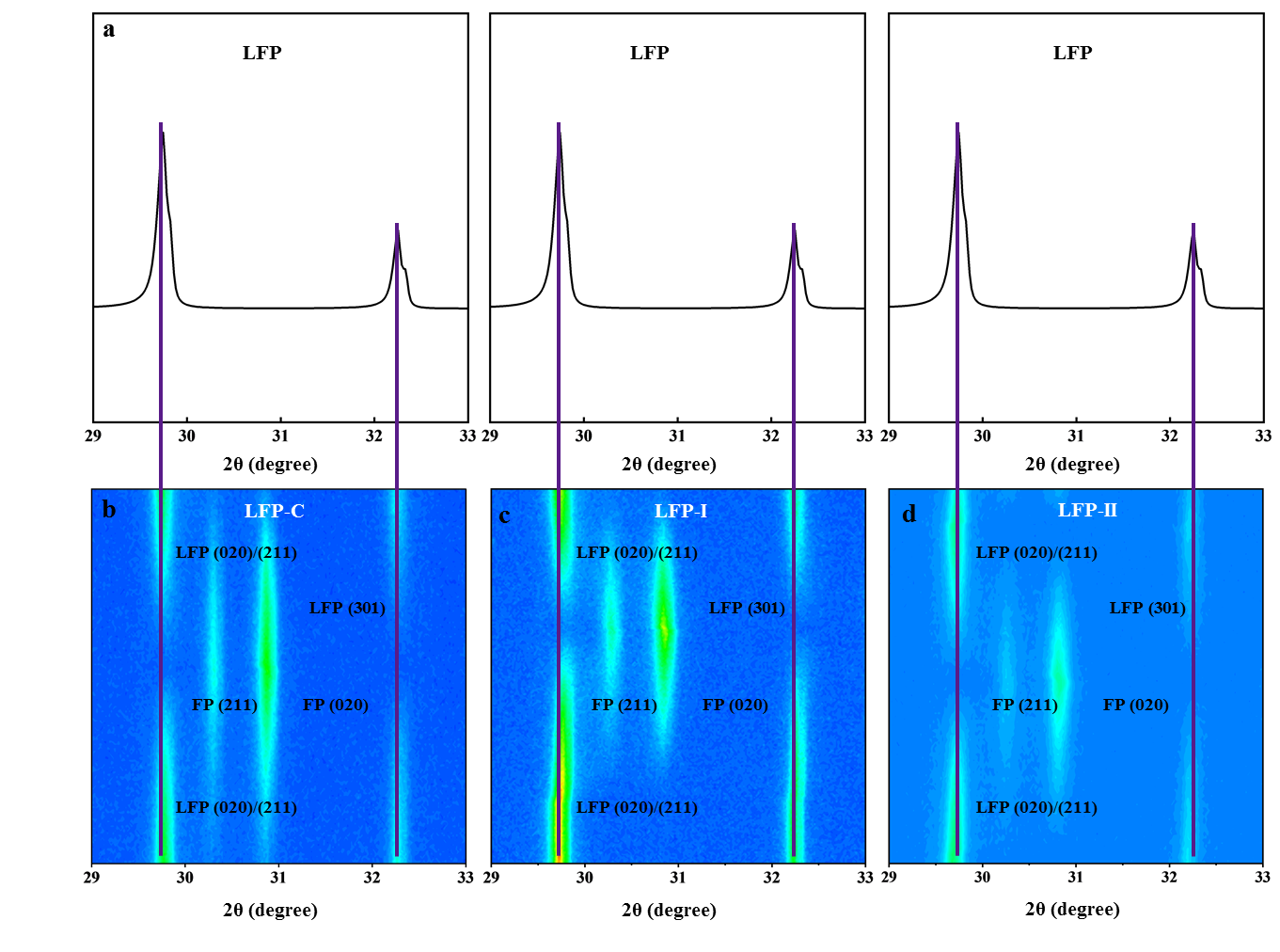


**Fig. S3** (**a**) the XRD spectrum of pristine LFP ;In situ XRD patterns of the evolution for (**b**) LFP-C, (**c**) LFP-I and (**d**) LFP-II

To further strengthen the reliability of our conclusions, we conducted additional comparative experiments under different activation conditions. In particular, for the charging-discharging-both-multi-step segmented indirect activation (LFP-II), the cells were cycled with charging at 0.4C, 0.3C, 0.2C, and 0.1C and discharging at the same sequence. In-situ XRD tests was employed to probe interfacial and structural evolution. In LFP-II, the diffraction peaks corresponding to the (020) and (211) planes remained relatively intense, while the FP (211) peak was weak, indicating incomplete phase transformation and increased resistance to Li+ migration. Conversely, in LFP-I, the diffraction peak intensities of the (020) and (211) planes gradually weakened until disappearance, while the FePO_4_ peaks (e.g., (211)) progressively intensified, confirming a complete orthorhombic-to-monoclinic transition during the LiFePO_4_ → FePO_4_ conversion.


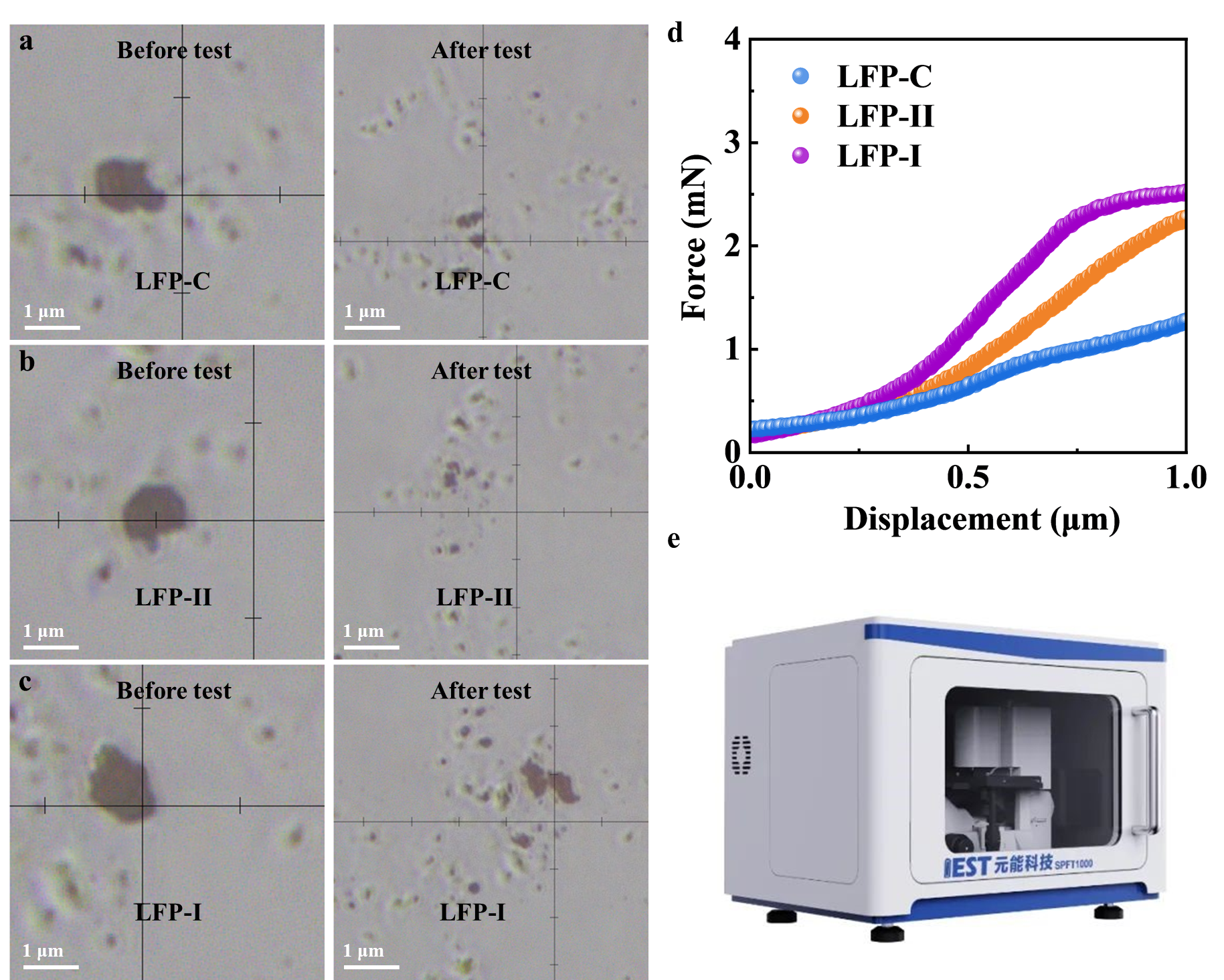


**Fig. S4** Optical photographs before and after single-particle stress testing for the (**a**) LFP-C, (**b**) LFP-II and (**c**) LFP-I; (**d**) Curve graph for the single-particle by (**e**) IEST, SPFT-2000


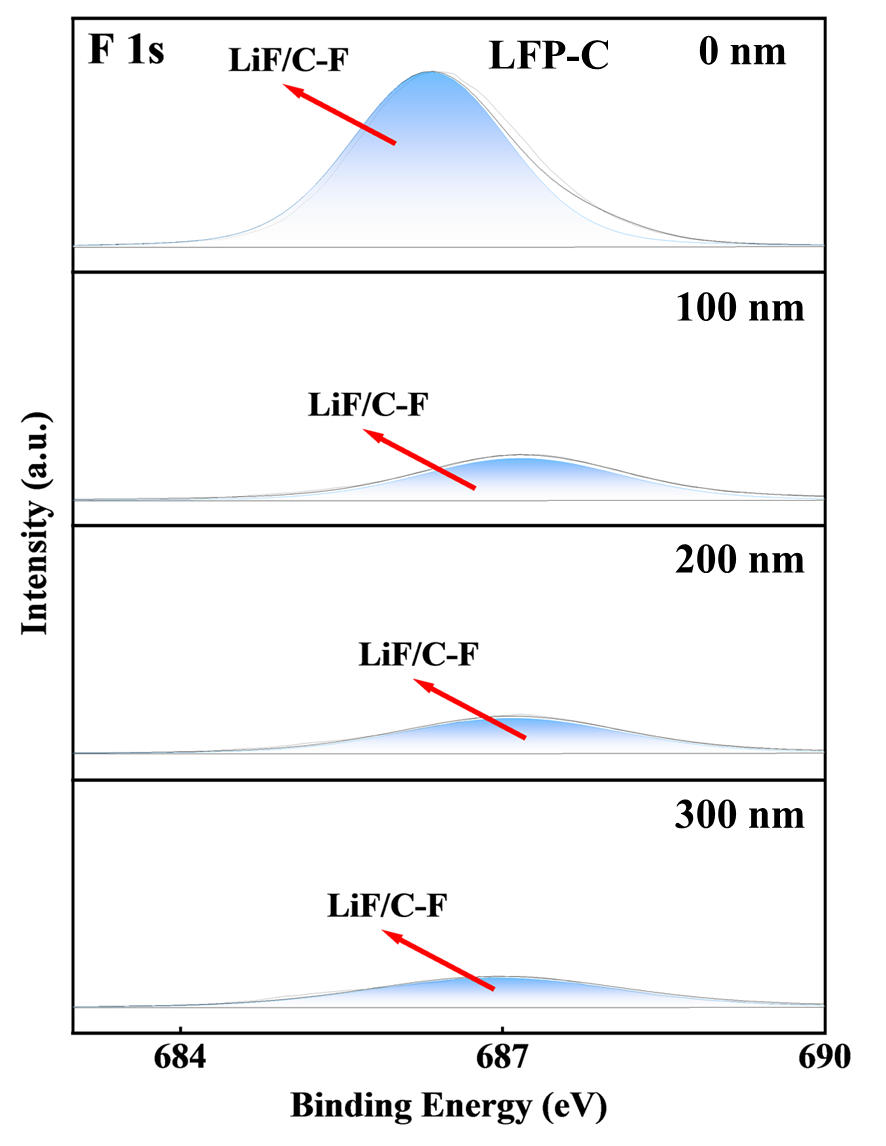


**Fig. S5** XPS spectra of F 1s for LFP-C in the different etching depths


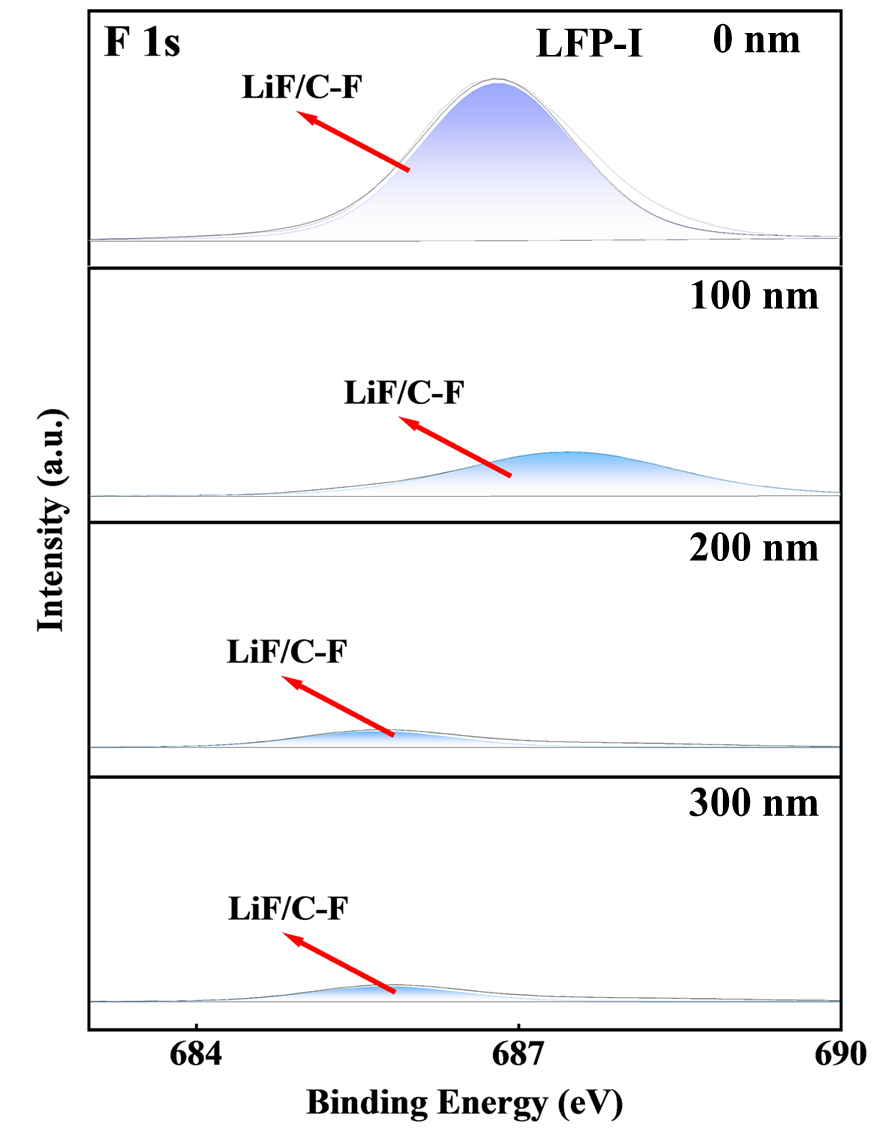


**Fig. S6** XPS spectra of F 1s for LFP-I in the different etching depths


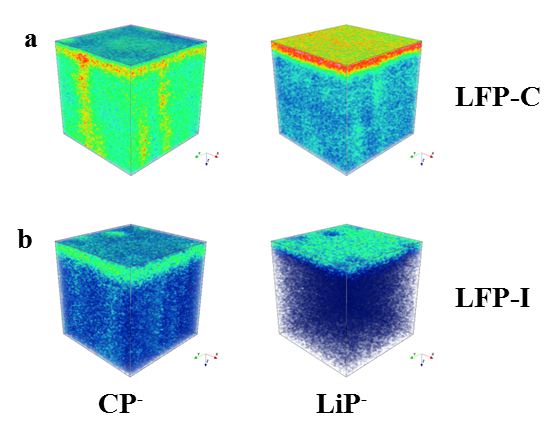


**Fig. S7** 3D compositions (CP^–^ and LiP^–^) for (**a**) LFP-C and (**b**) LFP-I; 3D compositions


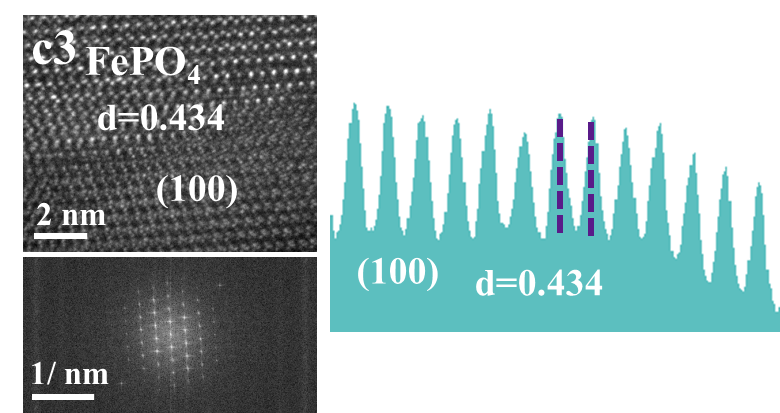


**Fig. S8** HR-TEM image of the selected area with the FFT image and lattice spacing diagram for LFP-C

**
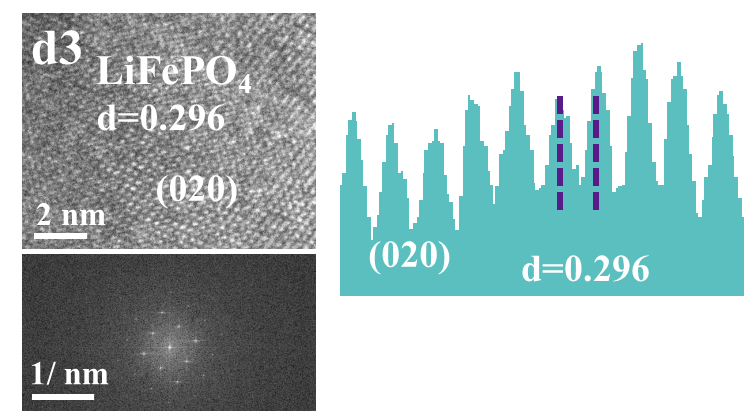
**

**Fig. S9** HR-TEM image for selecting the area with the FFT image and the lattice spacing diagram for LFP-I


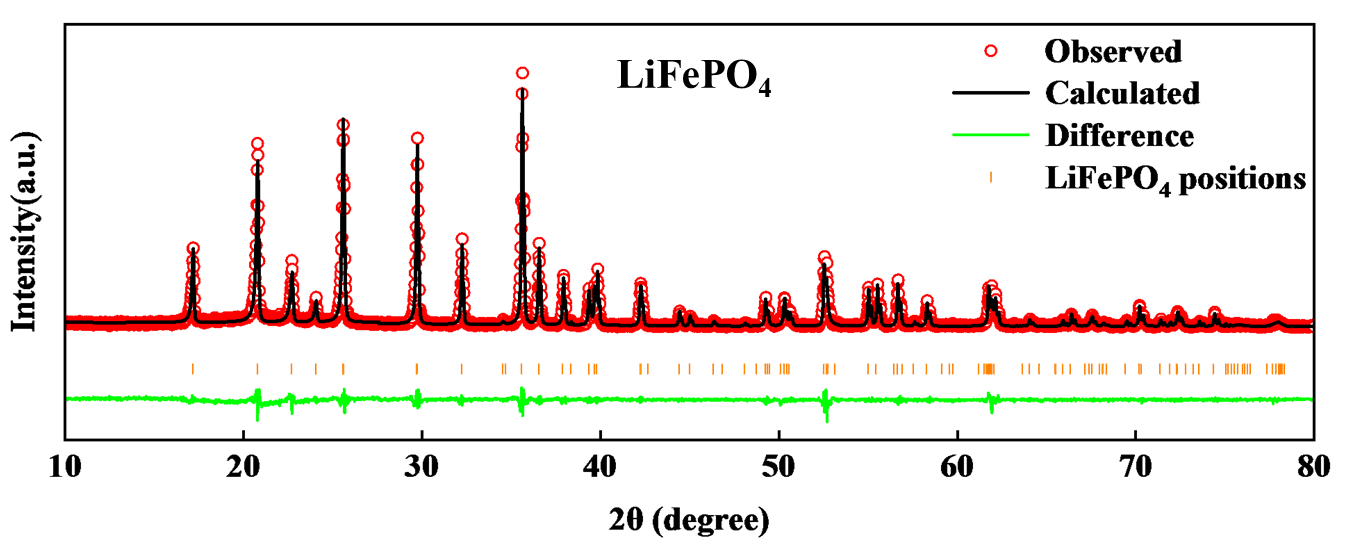


**Fig. S10** Rietveld reﬁnement results of pristine LiFePO_4_

**Table S1** The corresponding results of the Rietveld refinement

| Sample | R_wp_ | R_p_ | LiFePO_4_ | FePO_4_ | Chi 2 |
| --- | --- | --- | --- | --- | --- |
| LiFePO_4_ | 8.48% | 9.57% | / | / | 2.34 |
| LFP-C | 8.65% | 9.17% | 82% | 18% | 2.67 |
| LFP-I | 9.43% | 9.89% | 91% | 9% | 2.98 |


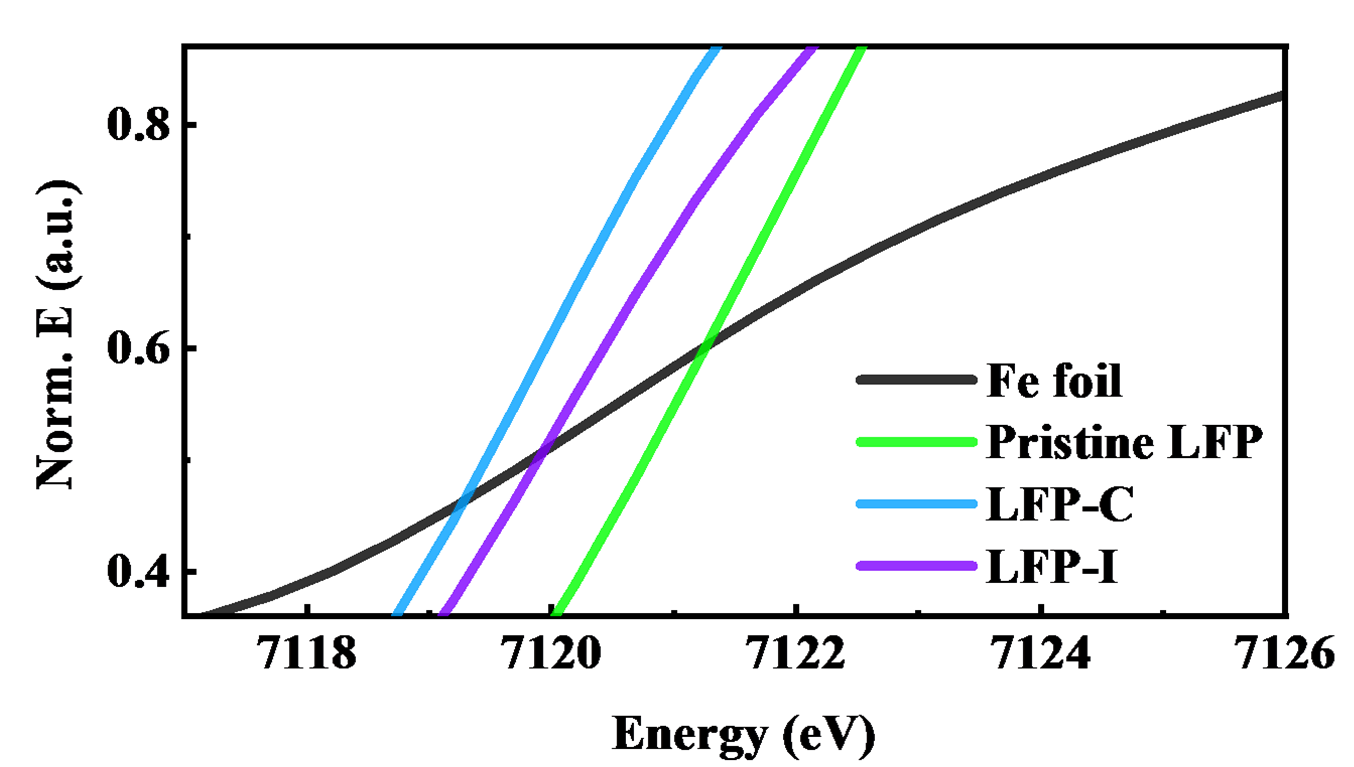


**Fig. S11** XANES spectra of the Fe K-edge before and after cycling with the corresponding local magnification

**
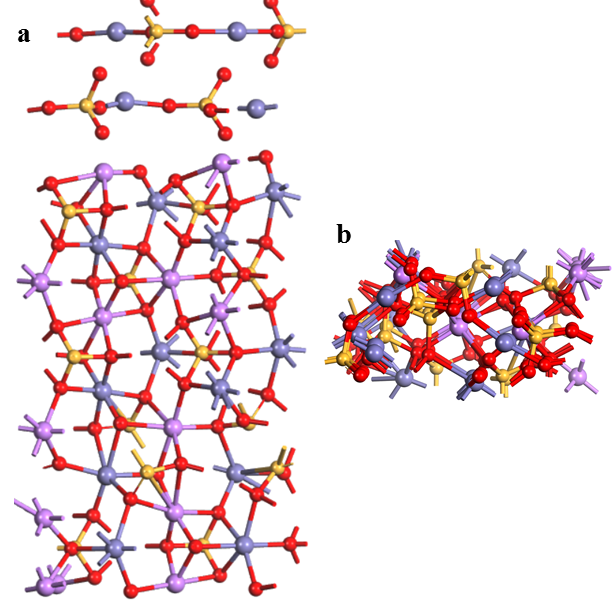
**

**Fig. S12** Density functional theory calculations. Crystalline structure and local morphology in (**a**) front and (**b**) overhead views for LFP-C

**
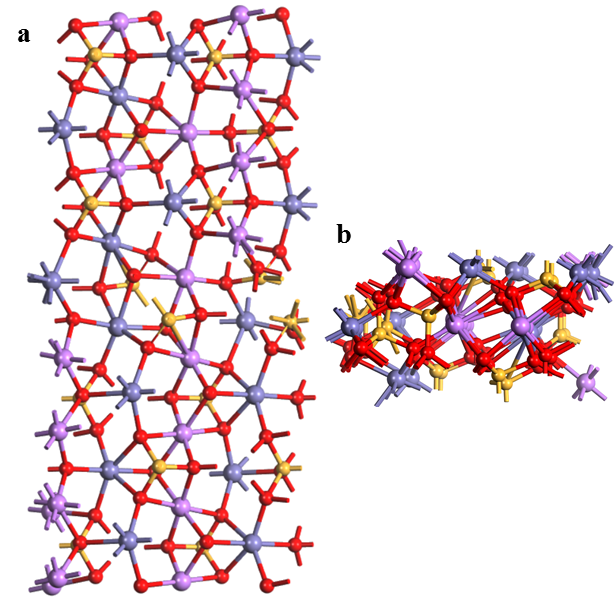
**

**Fig. S13** Density functional theory calculation. Crystalline structure and local morphology in (**a**) front and (**b**) overhead views for LFP-I

**
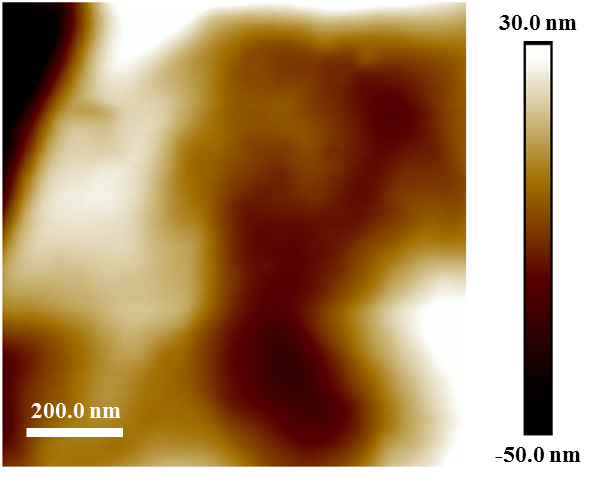
**

**Fig. S14** AFM analysis of 2D height image of LFP-C


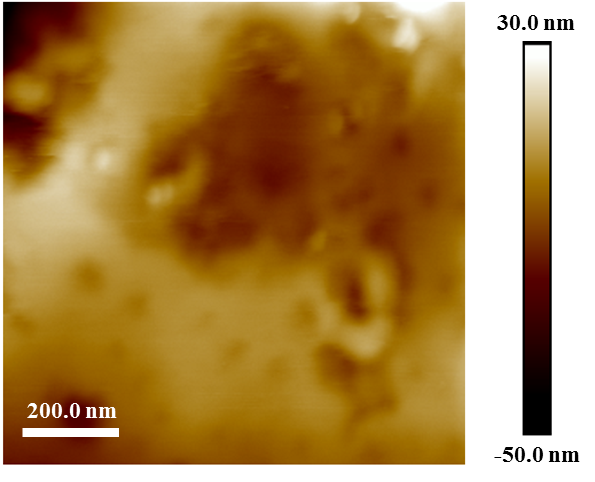


**Fig. S15** AFM analysis of 2D height image of LFP-I


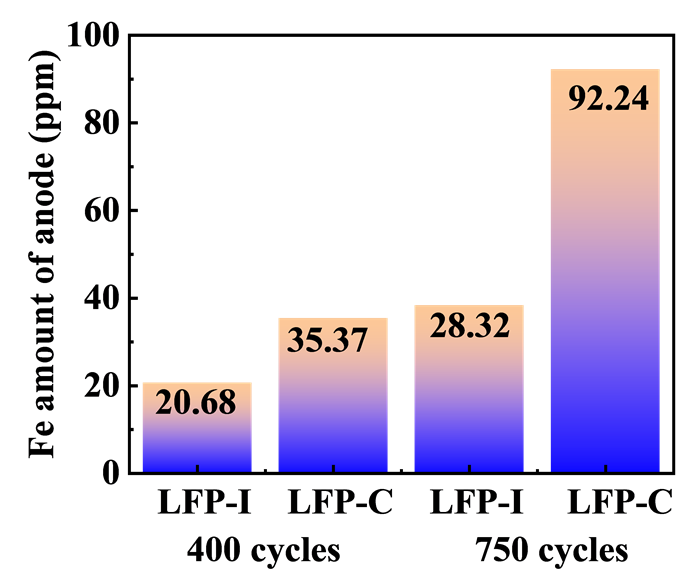


**Fig. S16** Fe dissolution of ICP-OES results for LFP-C and LFP-I after 400/650 cycles

Once Fe ions dissolve from the LFP cathode and migrate to the graphite anode, they can be electrochemically reduced under reductive potentials during charging (Fe^2+^ + 2e⁻ → Fe^0^). The deposition potential of Fe^2+^ is close to that of lithium plating, meaning that Fe reduction competes with Li nucleation at the anode surface. This competitive deposition lowers the effective lithium nucleation overpotential and introduces heterogeneous nucleation sites. Localized Fe deposits then disrupt the uniformity of the SEI, induce non-uniform Li^+^ flux, and catalyze further electrolyte decomposition.

In addition, Fe^3+^ species can participate in parasitic redox reactions at the SEI/electrolyte interface, generating radical intermediates that accelerate solvent decomposition and destabilize the SEI. Together, these redox processes explain how dissolved Fe species act as both catalytic and structural destabilizers at the negative electrode.

The observations with quantitative ICP-OES measurements to provide direct evidence of Fe dissolution and deposition behavior. After 400 cycles, the Fe content deposited on the graphite anode of LFP-C was measured as 35.37 ppm, while it was 20.68 ppm for LFP-I. Specifically, after 650 cycles, the dissolved Fe content in the electrolyte of LFP-C was as high as 92.24 ppm, while in LFP-I it was only 28.32 ppm, demonstrating a significant suppression of Fe dissolution in the latter case. These results provide direct quantitative confirmation that Fe dissolution and accumulation are much more severe in LFP-C.


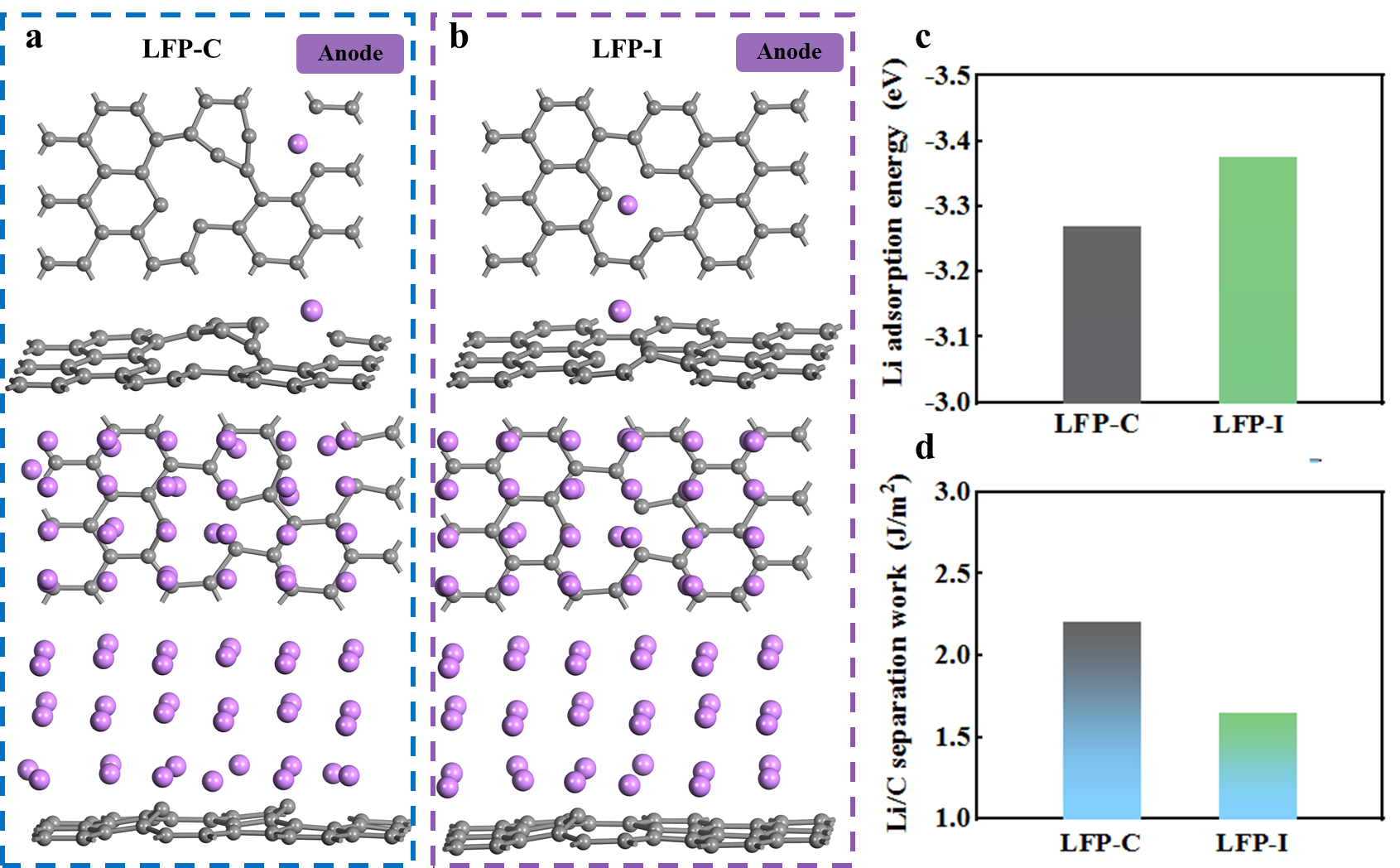


**Fig. S17** Crystal model for (**a**) LFP-C and (**b**) LFP-I on the anode electrode side; (**c**) Li adsorption energy; (**d**) Li /C separation work

Theoretical calculations assessed the interfacial binding characteristics. The lithium ion adsorption energy of LFP-I is higher than those of LFP-C, thereby accelerating the migration rate of Li+ ions at the interface. There are more defects at the LFP-C interface, which significantly affects the Li^+^ migration path, resulting in the loss of active Li. The separation work is also known as the adhesion work. The larger the value of the separation work, the stronger the interfacial binding ability. LFP-C binds lithium ions more firmly, indicating that higher energy is required for lithium lithiation/delithiation, resulting in a deterioration of performance. Furthermore, the structure of LFP-I is more ordered, LFP-I presents a more open framework, which is conducive to rapid lithiation/delithiation.


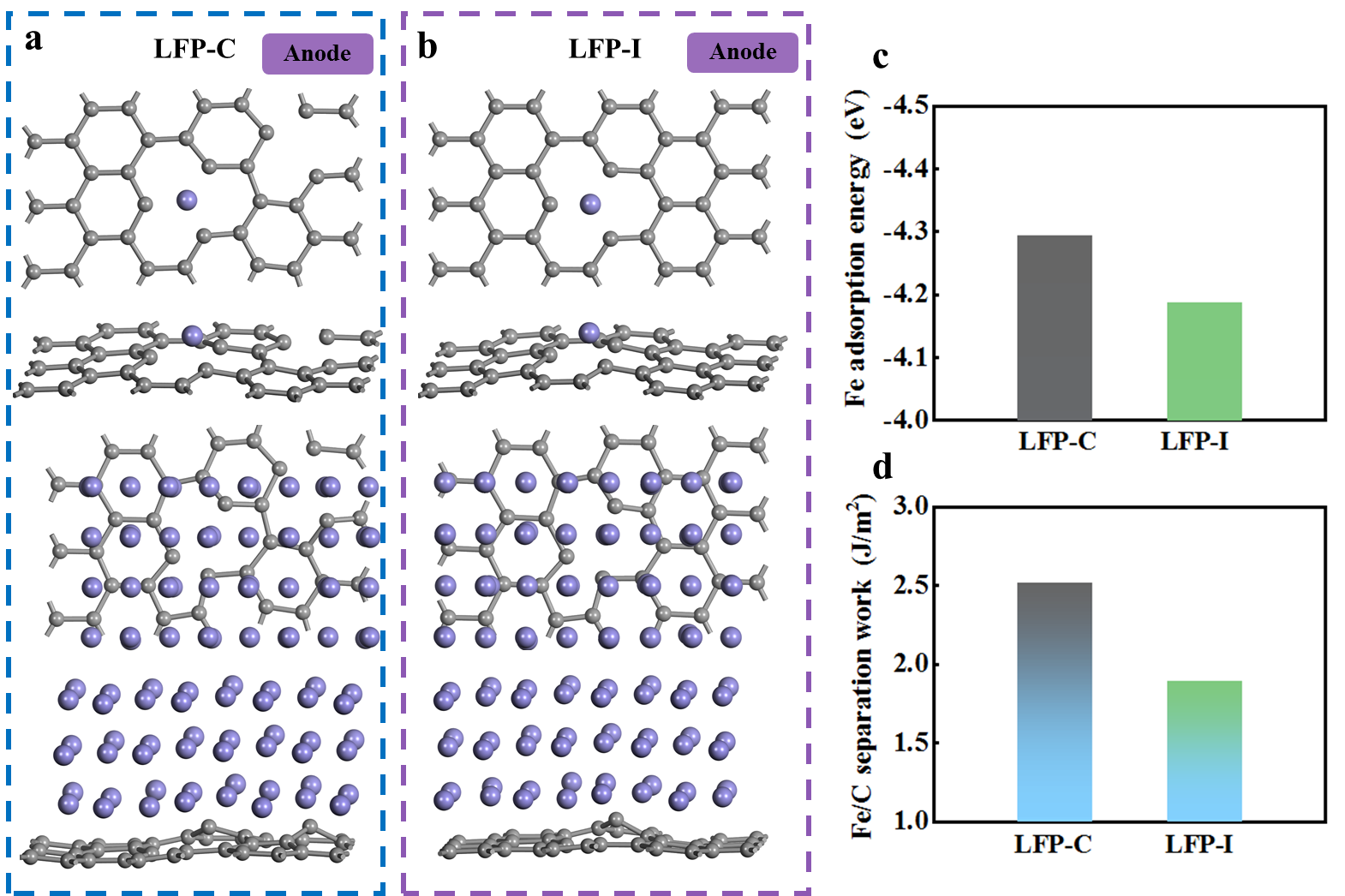


**Fig. S18** Crystal model for (**a**) LFP-C and (**b**) LFP-I on the anode electrode side; (**c**) Fe adsorption energy; (**d**) Fe/C separation work

The adsorption energy of Fe on the carbon substrate is higher in LFP-C, which means that Fe atoms are more strongly retained on the graphite anode surface. This strong binding leads to more persistent Fe deposition, resulting in local inhomogeneity, hindered lithium-ion transport, and accelerated electrode degradation. By contrast, the lower adsorption energy in LFP-I reflects weaker Fe–C interfacial binding, which is consistent with easier detachment of Fe species and the absence of excessive accumulation at the anode surface. This weaker interaction is also associated with a more open and uniform interfacial structure, which facilitates lithium-ion migration and contributes to improved electrochemical stability. Meanwhile, the LFP-C has a high separation work and requires more energy to separate Fe from the carbon interface. This further proves that Fe dissolves on the cathode electrode side and is difficult to detach from the anode electrode for LFP-C. This is obviously not conducive to electron conduction and causes the electrode to damage. The separation work of LFP-I is low and the interfacial binding is weak, which may provide a more flexible interfacial lithium-ion transport channel.


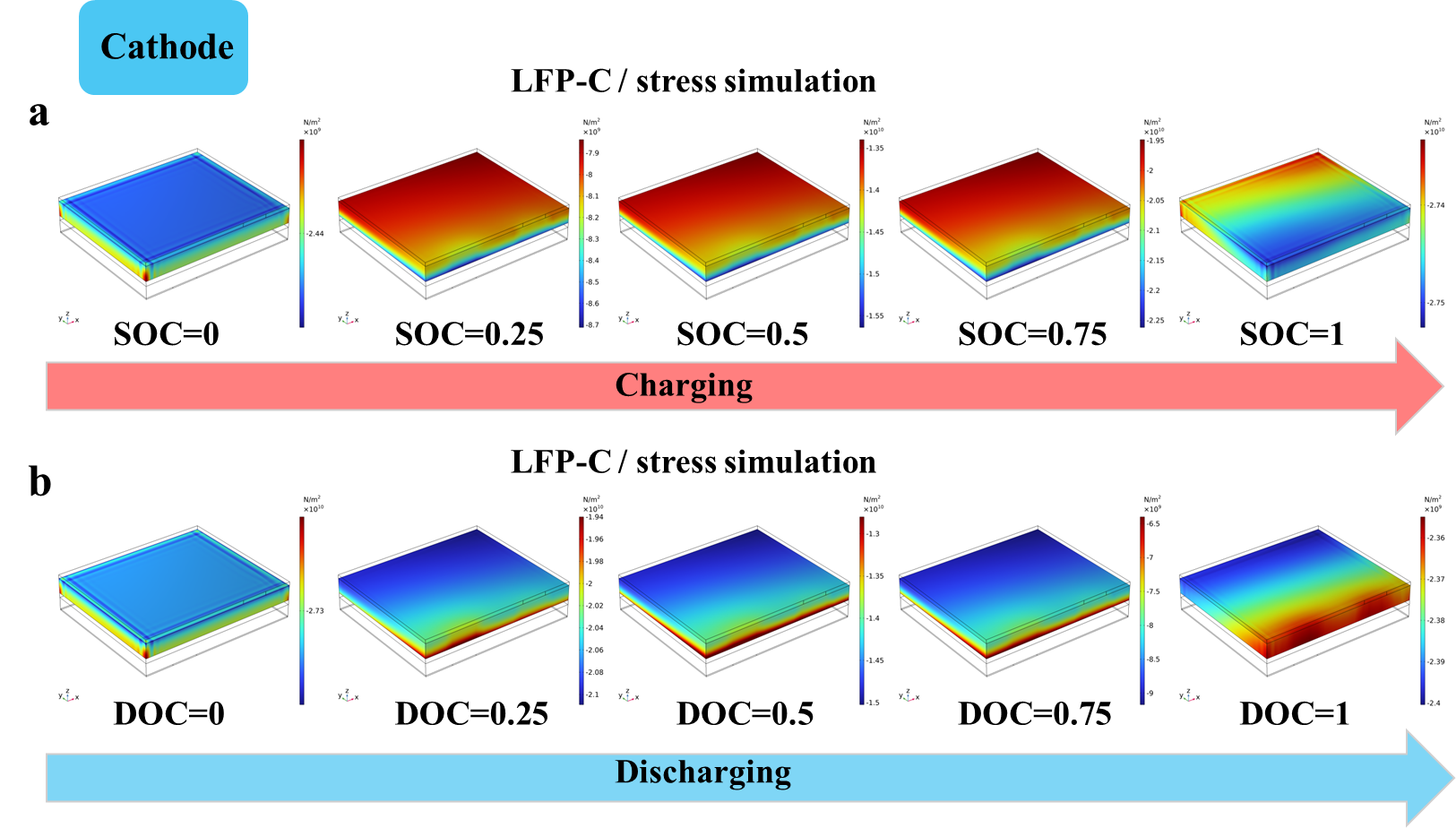


**Fig. S19** The stress simulation diagram of LFP-C for (**a**) charging progress and (**b**) discharging progress at the cathode


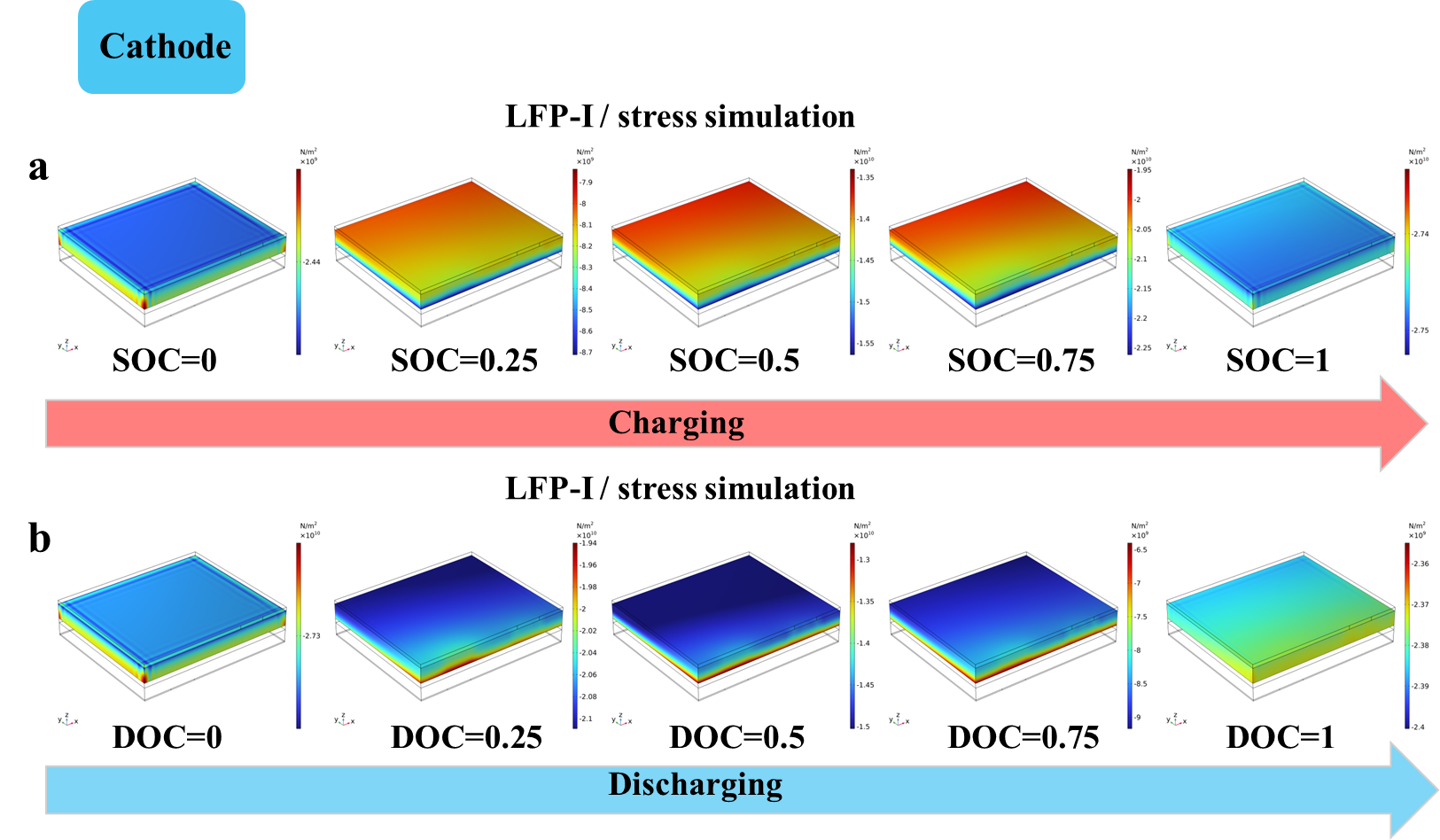


**Fig. S20** The stress simulation diagram of LFP-I for (**a**) charging progress and (**b**) discharging progress at the cathode


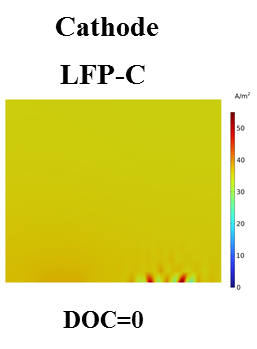


**Fig. S21** Current density of the LFP-C for DOC =0 in the cathode


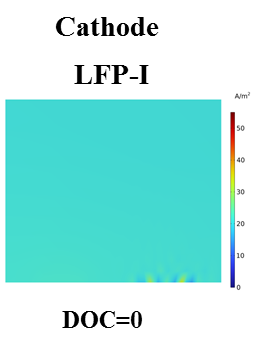


**Fig. S22** Current density of LFP-I for DOC =0 in the cathode


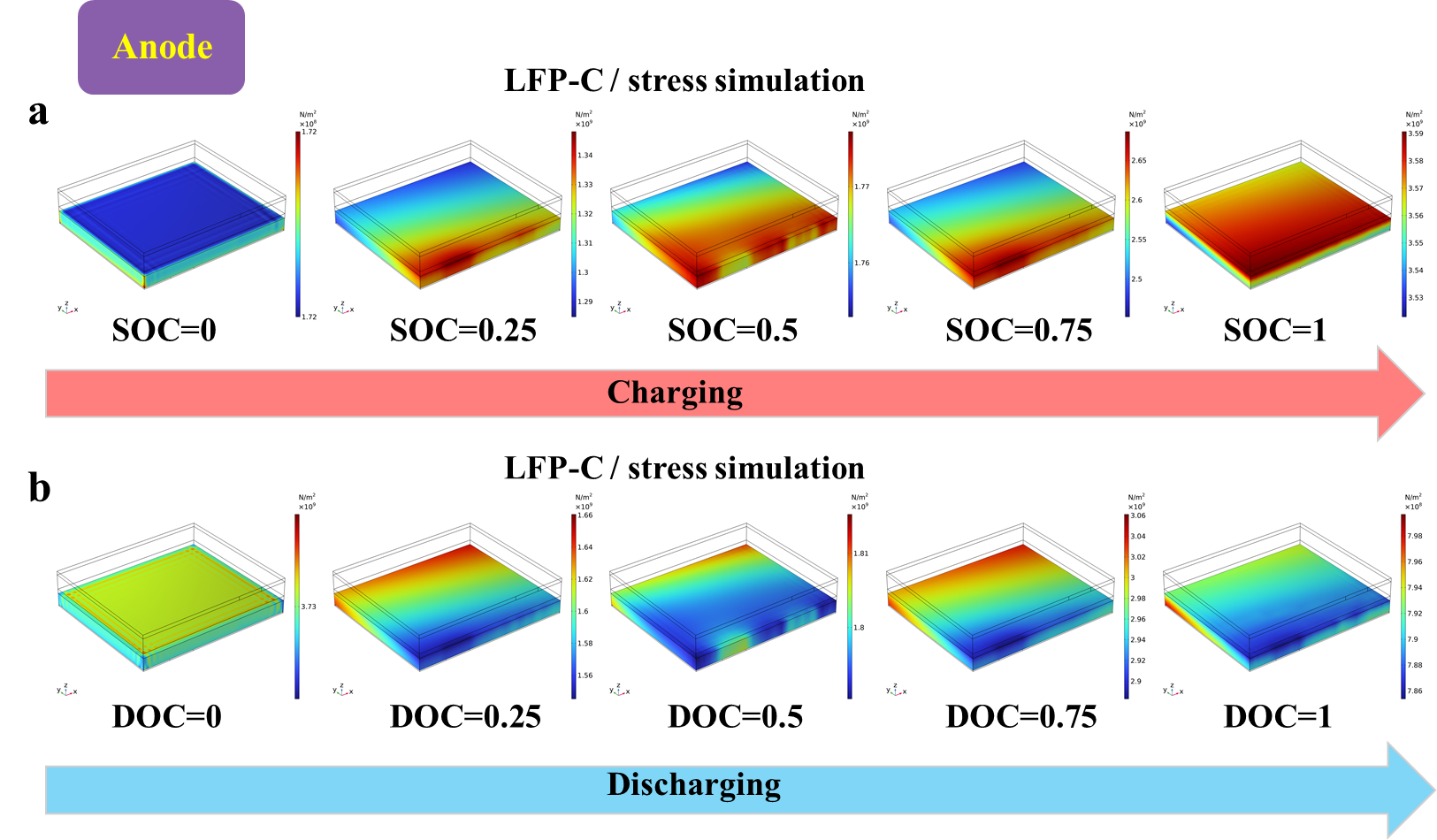


**Fig. S23** The stress simulation diagram of LFP-C for (**a**) charging progress and (**b**) discharging progress in the anode


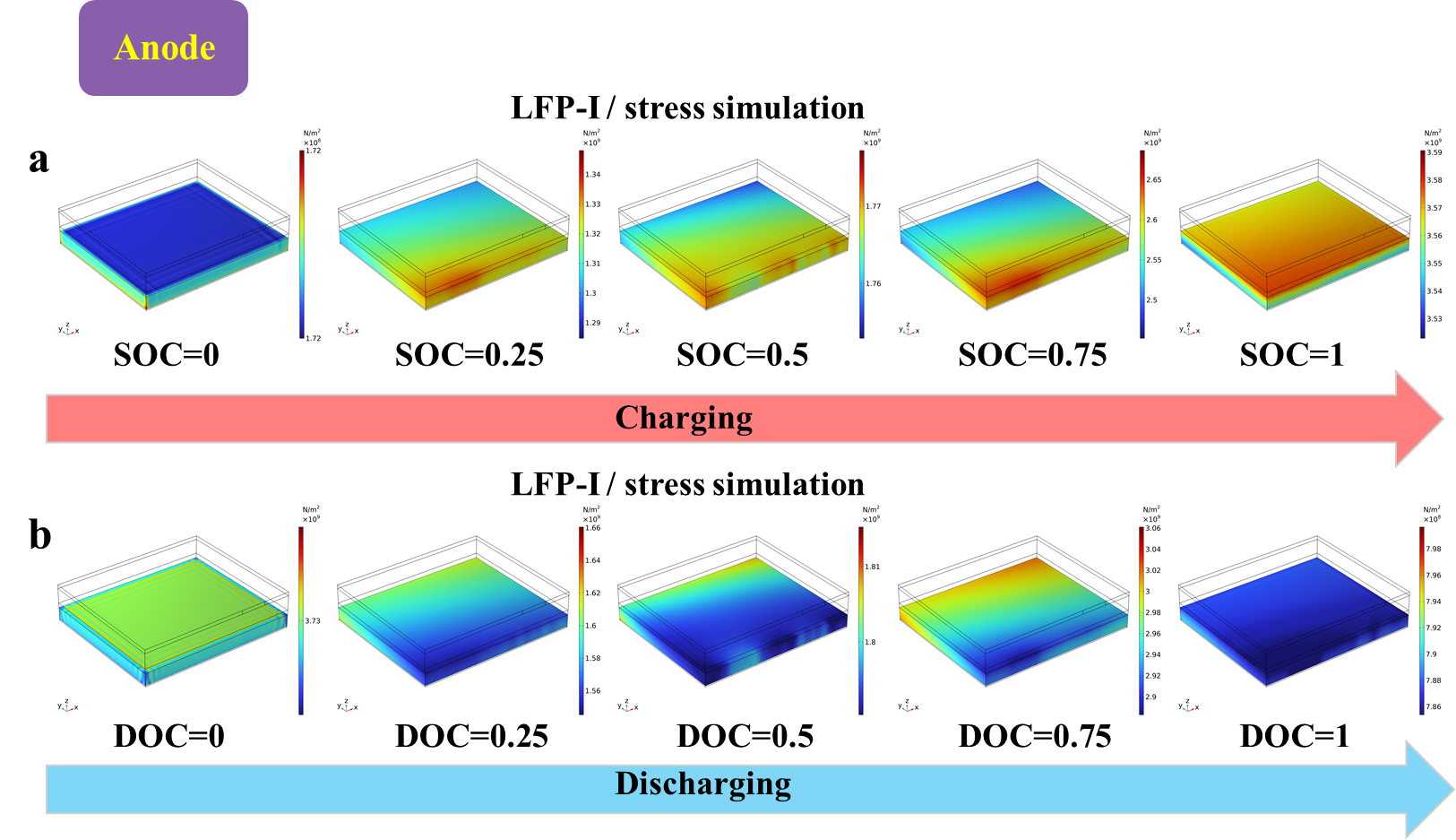


**Fig. S24** The stress simulation diagram of LFP-I for (**a**) charging progress and (**b**) discharging progress in the anode


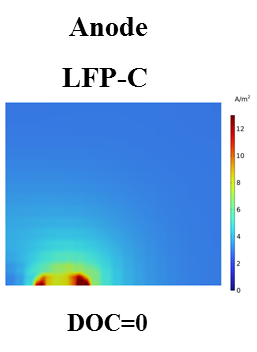


**Fig. S25** Current density of LFP-C for DOC =0 in the anode


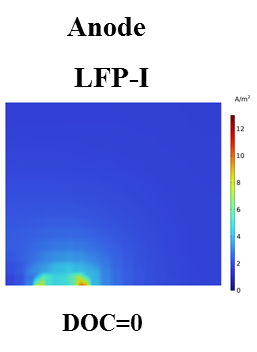


**Fig. S26** Current density of LFP-I for DOC =0 in the anode


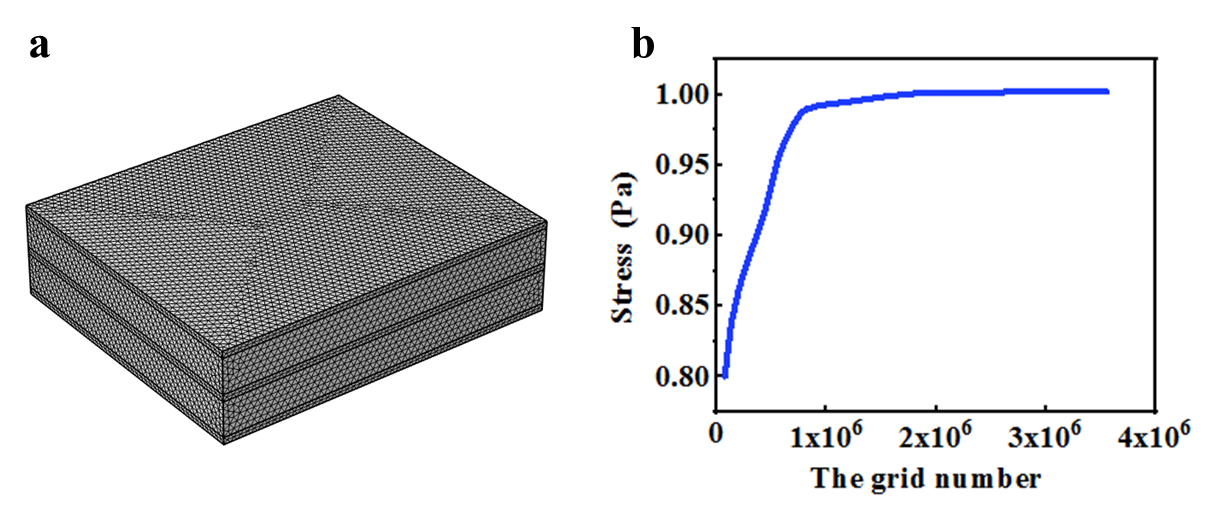


**Fig. S27** (**a**) The number of grids model; (**b**) Stress variation

The verification of grid independence for the stress calculations demonstrate that as the number of grids increases, the calculated stress values gradually converge and stabilize, showing that the numerical solution becomes insensitive to further mesh refinement. This confirms that the chosen mesh density is sufficient for accurate simulations and the influence of grid size on the final results can be safely neglected.
